# Lipidomic Characterization and Antioxidant Activity of Macro- and Microalgae Blend

**DOI:** 10.3390/life13010231

**Published:** 2023-01-13

**Authors:** Francisca Marques, Diana Lopes, Tiago Conde, Tânia Melo, Joana Silva, Maria Helena Abreu, Pedro Domingues, Maria Rosário Domingues

**Affiliations:** 1 CESAM—Centre for Environmental and Marine Studies, Department of Chemistry, University of Aveiro, Santiago University Campus, 3810-193 Aveiro, Portugal; 2 Mass Spectrometry Centre, LAQV-REQUIMTE, Department of Chemistry, University of Aveiro, Santiago University Campus, 3810-193 Aveiro, Portugal; 3 Allmicroalgae Natural Products S.A., R&D Department, Rua 25 de Abril 19, 2445-287 Pataias, Portugal; 4 ALGAplus—Production and Trading of Seaweed and Derived Products Ltd., 3830-196 Ílhavo, Portugal

**Keywords:** algae blend, microalgae, lipids, lipidomics, antioxidant activity, mass spectrometry

## Abstract

Macro- and microalgae are currently recognized sources of lipids with great nutritional quality and attractive bioactivities for human health promotion and disease prevention. Due to the lipidomic diversity observed among algae species, giving rise to different nutritional and functional characteristics, the mixture of macro- and microalgae has the potential to present important synergistic effects resulting from the complementarity among algae. The aim of this work was to characterize for the first time the lipidome of a blend of macro- and microalgae and evaluate the antioxidant capacity of its lipid fraction. Fatty acids were profiled by GC-MS, the polar lipidome was identified by high resolution LC-MS, and ABTS^+•^ and DPPH^•^ assays were used to assess the antioxidant potential. The most abundant fatty acids were oleic (18:1 *n*-9), α-linolenic (18:3 *n*-3), and linoleic (18:2 *n*-6) acids. The lipid extract presented a beneficial *n*-6/*n*-3 ratio (0.98) and low values of atherogenic (0.41) and thrombogenic indices (0.27). The polar lipidome revealed 462 lipid species distributed by glycolipids, phospholipids, and betaine lipids, including some species bearing PUFA and a few with reported bioactivities. The lipid extract also showed antioxidant activity. Overall, the results are promising for the valorization of this blend for food, nutraceutical, and biotechnological applications.

## 1. Introduction

A trend towards algae (macroalgae and microalgae) has been experienced in recent years by western countries, which is mirrored in the increase in the number of companies producing algae, particularly in Europe [1]. Algae are one of the most important marine and fresh water resources, with the potential to support carbon neutrality, the transition fostered by innovation towards healthy and sustainable food systems, and the green circular bioeconomy [2,3,4]. In this context, algae and algae-based products are progressively being used as food and food ingredients, and also the integral use and valorization of algae have been boosted, which presupposes the stepwise separation of relevant algae components for high-value applications, including functional food, cosmetics, and pharmaceutics [5]. Among its different components, algae have earned rising interest as sources of lipids, not only for biodiesel production, but also as a sustainable alternative to fish lipids, with great nutritional quality and attractive bioactivities [6,7].

The lipid fraction of macroalgae, also known as seaweeds, accounts for 1 to 8% of dry matter [8,9,10], while in microalgae, the lipid content varies within 10 to 50% of dry matter, but it can reach higher values (≥70%), depending on the growth conditions [11]. Several works have already targeted the lipid signatures of diverse algae species, including the macroalgae *Ulva rigida* [9,12] and *Fucus vesiculosus* [13], and the microalgae *Chlorella vulgaris* [14,15], from the fatty acid composition to the in-depth profiling of lipid classes and species. Each algae shows a specific lipidome that comprises a particular composition in fatty acids and in polar lipids—glycolipids, phospholipids, and betaine lipids [7,16]. Despite the substantial variety of profiles, lipidomic studies revealed the occurrence of noteworthy lipid species, namely, the interesting content of polyunsaturated fatty acids (PUFA) and particularly of long chain *n*-3 PUFA, such as α-linolenic (18:3 *n*-3) and eicosapentaenoic (20:5 *n*-3) acids [17], with well-recognized health benefits mainly against non-communicable diseases [18]. Interestingly, algae PUFA occur mainly esterified in polar lipids [12,13,14], which seems to increase the bioavailability of these healthy fatty acids [19]. Polar lipid species also display intrinsic health-promoting properties (e.g., antioxidant and anti-inflammatory activities), representing great potential for the prevention and mitigation of cardiovascular and other chronic diseases [20,21].

Algae have also drawn attention as a reservoir of antioxidant phytochemicals, in line with the demand for novel antioxidants that is driving the exploration and bioprospection of marine resources [18]. Antioxidants play a critical role in slowing or preventing oxidative stress events that underlie numerous pathological conditions, principally chronic and degenerative diseases [22]. Furthermore, they can expand the shelf life of products susceptible to oxidation, such as food and cosmetics, preserving their quality for a longer time period [22]. Algae lipids are a promising eco-friendly alternative to synthetic antioxidants, such as butylated hydroxyanisole (BHA; E-320) or butylated hydroxytoluene (BHT; E-321) [17,23], in conformity with the request of the food and healthcare industries for natural ingredients, encouraged by consumer demand [24].

Due to the lipidomic diversity observed among different algae species, giving rise to dissimilar characteristics, the mixture of several algae species has the potential to present important synergistic effects resulting from the complementarity among algae, which is not possible to obtain with only one species [25]. Regarding algae blends, the market offer is limited to products that combine only macroalgae; therefore, a blend of macro- and microalgae is a completely innovative add-value conception. The precise identification of lipids from any algae blend can elucidate its nutritional and health value, as well as encourage the development of groundbreaking products based on these lipids for multiple applications [26]. Thus, in this work, we aimed to identify for the first time the lipidome of an innovative blend of macro- and microalgae made with *Chlorella vulgaris*, *Fucus vesiculosus*, and *Ulva rigida*. The polar lipidome was analyzed and characterized in detail using a lipidomic approach based on the hydrophilic interaction liquid chromatography coupled to electrospray ionization high resolution tandem mass spectrometry (HILIC-ESI-HR-MS/MS), and gas chromatography–mass spectrometry (GC-MS) allowed profiling of the total esterified fatty acids. To improve the exploitation and valorization of the blend’s total lipids, the antioxidant potential was screened using the most common spectrophotometric free-radical scavenging assays, i.e., ABTS and DPPH tests. Overall, with this work, we aim to demonstrate that the blend has additional values to be used in food, nutraceutical, and biotechnological applications, specifically at the lipid level.

## 2. Materials and Methods

### 2.1. Algae Material

Algae blend (Algaessence^®^; BLEND) biomass was provided by ALGAplus (Ílhavo, Portugal). Algaessence^®^ is a microalgae and macroalgae blend composed of organic *Chlorella vulgaris*, *Fucus vesiculosus*, and *Ulva rigida*. Macroalgae (*F. vesiculosus* and *U. rigida*) were produced by ALGAplus, and microalgae (*C. vulgaris*) was cultivated under autotrophic conditions by Allmicroalgae Natural Products (Pataias, Portugal).

### 2.2. Lipid Extraction

Total lipids extraction was performed according to a modified Bligh and Dyer method [25]. To 250 mg of the BLEND biomass (*n* = 5) was added 3.75 mL of a solvent mixture of methanol and dichloromethane (2:1, *v*/*v*), followed by homogenization in a vortex for 2 min and ultrasonication in an ultrasound bath for 1 min. After ice incubation on a rocking platform shaker (Stuart equipment, Cole-Parmer Ltd, UK) for 2 h, the suspension was centrifuged (Selecta JP Mixtasel, Abrera, Barcelona, Spain) for 10 min at 2000 rpm, and the organic phase was collected. To promote phase separation and wash the lipid extract, 1.25 mL of dichloromethane and 2.25 mL of Milli-Q water were added to the collected organic phase, centrifuged for 10 min at 2000 rpm, and the lower organic phase was recovered. Re-extraction steps using the same solvent proportions were repeated two more times. The organic phases collected were dried under a stream of nitrogen. Extracts were weighed to determine lipid content and stored at −20 °C. The results were expressed in g 100^−1^ of dry weight of sample (DW).

### 2.3. Fatty Acids Analysis by Gas Chromatography–Mass Spectrometry (GC–MS)

Fatty acid profiling was performed by gas chromatography–mass spectrometry (GC-MS) analysis of the fatty acid methyl esters (FAMEs). For that, FAMEs were prepared from the BLEND total lipid extract by alkaline transmethylation reaction using a methanolic solution of potassium hydroxide (2.0 M), according to the procedure previously detailed by Rey et al. [7]. A volume of 2.0 μL of a hexane solution containing FAMEs (*n* = 5) was injected in a GC-MS (Agilent Technologies 6890 N Network, Santa Clara, CA, USA) equipped with a DB-FFAP column with the following specifications: 30 m long, 0.32 mm internal diameter, and 0.25 μm film thickness (J & W Scientific, Folsom, CA, USA). The GC equipment was connected to an Agilent 5977B Mass Selective Detector operating with an electron impact mode at 70 eV and scanning range of 50–550 *m/z* in a one-second cycle in full scan mode acquisition. Helium was the carrier gas at a flow rate of 1.4 mL min^−1^. The injector and detector temperatures were 220 °C and 230 °C, respectively. The oven temperature was programmed as follows: 58 °C for 2 min, 25 °C min^−1^ to 160 °C, 2 °C min^−1^ to 210 °C, and 30 °C min^−1^ to 225 °C, and held for 20 min. Data acquisition was performed using GCMS5977B/Enhanced MassHunter. Data were analyzed using Agilent MassHunter Qualitative Analysis 10.0 software, and fatty acid identification was conducted by the retention time and MS spectrum comparison with the NIST chemical database library and confirmed with literature reports. The relative amount of each fatty acid was calculated by the percent relative area method, with proper normalization using internal standard methyl nonadecanoate (C19:0, Sigma-Aldrich, St. Louis, MO, USA), considering the sum of all relative areas of identified fatty acids.

Nutritional quality of the lipid fraction was assessed based on the fatty acid profile, through the PUFA/SFA and PUFA *n*-6/*n*-3 ratios, and by the atherogenicity (AI) and thrombogenicity (TI) indices, which were calculated according to the equations listed below.

(1) $$\mathrm{AI}=\frac{\mathrm{FA}\;12:0+(4\;\times \;\mathrm{FA}\;14:0)+\mathrm{FA}\;16:0\;}{\sum^{\;}\mathrm{MUFA}+\sum^{\;}\mathrm{PUFA}}$$

(2) $$\mathrm{TI}=\frac{\mathrm{FA}\;14:0+\mathrm{FA}\;16:0+\mathrm{FA}\;18:0\;}{\left(0.5\;\times \;\sum^{\;}\mathrm{MUFA}\right)+\left(0.5\;\times \;\sum^{\;}n-6\right)+\left(3\;\times \;\sum^{\;}n-3\right)+(\mathrm{PUFA}\;n-6/n-3)}$$

### 2.4. Polar Lipidome Analysis by Hydrophilic Interaction Liquid Chromatography-High-Resolution Tandem Mass Spectrometry (HILIC-HR-MS/MS)

The polar lipidome was determined by hydrophilic interaction liquid chromatography (HILIC) in an Ultimate 3000 Dionex (Thermo Fisher Scientific, Bremen, Germany) with an autosampler coupled to the Q-Exactive^®^ hybrid quadrupole Orbitrap mass spectrometer (Thermo Fisher, Scientific, Bremen, Germany), according to the methodology previously described [15]. The solvent system used consisted of two mobile phases, A and B, which were respectively composed of acetonitrile:methanol:water (2:1:1, *v*/*v*/*v*) and acetonitrile:methanol (3:2, *v*/*v*), both containing 5 mM ammonium acetate. The gradient program applied was as follow: 5% A (0–2 min), 5–48% A (2–10 min), 48–65% A (10–15 min), 65% A (15–17 min), returning to the initial conditions in 3 min and held for 10 min.

A mixture containing 40 μg of the BLEND total lipid extract (in 20 µL of dichloromethane), 72 μL of starting eluent (95% B and 5% A), and 8 μL of an internal standards mixture (dMPC-0.04 µg, dMPE-0.04 µg, dPPI-0.08 µg, dMPG-0.024 µg, dMPS-0.08 µg, dMPA-0.16 μg, LPC-0.04 µg, SM d18:1/17:0-0.04 µg, Cer(17:0/d18:1)-0.08 μg, CL(14:0)_4_-0.16 µg) was prepared. Then, a 5 μL aliquot of the sample mixture (*n* = 5) was injected into the microbore Ascentis^®^ Express column (10 cm × 2.1 mm, 2.7 µm; Sigma-Aldrich) with a flow rate of 200 μL min^−1^ at 35 °C. The mass spectrometer was operated simultaneously in positive (electrospray voltage 3.0 kV) and negative (electrospray voltage—2.7 kV) modes, configured as follows: high resolution of 70,000, capillary temperature of 350 °C, sheath gas flow of 20 U, automatic gain control (AGC) target of 1 × 10^6^, *m*/*z* range of 400–1600, 2 microscans, and maximum inject time (IT) of 100 ms. In MS/MS experiments, the following configuration was set: high resolution of 17,500, AGC target of 1 × 10^5^, 1 microscan, and maximum IT of 50 ms. Cycles of one full-scan mass spectrum and ten data-dependent MS/MS scans were continuously repeated throughout the experiments, with a dynamic exclusion of 60 s, intensity threshold of 2 × 10^4^, and normalized collision energy ranged between 25, 30, and 35 eV. Data acquisition was accomplished using the Xcalibur v3.3 software (Thermo Fisher Scientific, USA), which was also used to analyze the acquired spectra in order to identify the polar lipid species.

The identification was performed based on the approach previously described [27]. Briefly, lipid species was assigned according to the retention time of internal standards and accurate mass measurements (error of <5 ppm), and the interpretation of well-known fragmentation patterns provided structural information and validated the identification of the most lipid species. To assist in the identification, MZmine v2.42 software was used to filter the raw LC-MS data, peak detection (intensity threshold of 1 × 10^4^), peak processing, and assignment against an in-house database.

### 2.5. ABTS^+•^ Scavenging Assay

The antioxidant scavenging activity against the 2,2′-azinobis-3-ethylbenzthiazoline-6-sulphonic acid radical cation (ABTS^+•^) was evaluated as performed in other studies [8,17]. A volume of 150 μL of the lipid extract (31.25–250 μg mL^−1^ in ethanol, *n* = 3) or 150 μL of the Trolox standard solution (5–37.5 μmol L^−1^ in ethanol) were mixed with 150 μL of an ABTS^+•^ working solution in ethanol (3.5 mmol L^−1^; absorbance ≈ 0.9, 734 nm). The mixture was incubated for 120 min, and the absorbance was measured at 734 nm every 5 min (Multiskan GO 1.00.38, Thermo Scientific, Hudson, NH, USA). Control lipid extracts were prepared by replacing the ABTS^+•^ solution with ethanol. The antioxidant activity, expressed as a percentage of inhibition of the ABTS^+•^, was determined according to Equation (3), where ${\mathrm{Abs}}_{{\mathrm{ABTS}}^{+•}}$ is the absorbance of the ABTS^+•^ and ${\mathrm{Abs}}_{\mathrm{sample}-\mathrm{control}}$ is the difference between the absorbance of the sample and the absorbance of the control.

(3) $$\mathrm{Inhibition}\;(\%)=\frac{{\mathrm{Abs}}_{{\mathrm{ABTS}}^{+•}\;-}\;{\mathrm{Abs}}_{\mathrm{sample}-\mathrm{control}}}{{\mathrm{Abs}}_{{\mathrm{ABTS}}^{+•}}}\times \;100$$

The lipid extract concentration capable of scavenging 50% (IC_50_) of ABTS^+•^ after 120 min of reaction was calculated by linear regression plotting of the concentration of lipid extract versus the percentage of the inhibition curve. The activity was expressed as Trolox Equivalents (TE, μmol Trolox g^−1^ of sample), according to Equation (4).

(4) $$\mathrm{TE}=\frac{{\mathrm{IC}}_{50}\;\mathrm{Trolox}\;(\mu \mathrm{mol}\;L-1)}{{\mathrm{IC}}_{50}\;\mathrm{of}\;\mathrm{sample}\;(\mu g\;\mathrm{mL}-1)}\times \;1000$$

Calibration curve for ABTS test is provided in Supplementary Figure S1.

### 2.6. DPPH^•^ Scavenging Assay

The antioxidant scavenging activity against the 2,2-diphenyl-1-picrylhydrazyl radical (DPPH^•^) was determined using a previously described method [8,17]. A volume of 150 μL of the lipid extracts (31.25–250 μg mL^−1^ in ethanol, *n* = 3) or 150 μL of the Trolox standard solution (5–37.5 μmol L^−1^ in ethanol) were mixed with 150 μL of a DPPH^•^ working solution in ethanol (250 μmol L^−1^; absorbance ≈ 0.9, 517 nm). After incubating the mixture for 120 min, the absorbance was measured at 517 nm every 5 min (Multiskan GO 1.00.38, Thermo Scientific, Hudson, NH, USA). Control lipid extracts were prepared by replacing the DPPH^•^ solution with ethanol. The antioxidant activity, expressed as a percentage of inhibition of the DPPH^•^, was determined according to Equation (5), where ${\mathrm{Abs}}_{{\mathrm{DPPH}}^{•}}$ is the absorbance of the ABTS^+•^ and ${\mathrm{Abs}}_{\mathrm{sample}-\mathrm{control}}$ is the difference between the absorbance of the sample and the absorbance of the control.

(5) $$\mathrm{Inhibition}\;(\%)=\frac{{\mathrm{Abs}}_{{\mathrm{DPPH}}^{•}}{\;-\;\mathrm{Abs}}_{\mathrm{sample}-\mathrm{control}}}{{\mathrm{Abs}}_{{\mathrm{DPPH}}^{•}}}\times \;100$$

The lipid extract concentration capable of scavenging 35% (IC_35_) and 20% (IC_20_) of DPPH^•^ after 120 min of reaction was calculated by linear regression plotting of the concentration of lipid extract versus the percentage of the inhibition curve. The activity was expressed as Trolox Equivalents (TE, μmol Trolox g^−1^ of sample), according to Equation (6) for IC_35_ and Equation (7) for IC_20_

(6) $$\mathrm{TE}=\frac{{\mathrm{IC}}_{35}\;\mathrm{Trolox}\;(\mu \mathrm{mol}\;L^{-1})}{{\mathrm{IC}}_{35}\;\mathrm{of}\;\mathrm{sample}\;(\mu g\;{\mathrm{mL}}^{-1})}\times \;1000$$

(7) $$\mathrm{TE}=\frac{{\mathrm{IC}}_{20}\;\mathrm{Trolox}\;(\mu \mathrm{mol}\;L^{-1})}{{\mathrm{IC}}_{20}\;\mathrm{of}\;\mathrm{sample}\;(\mu g\;{\mathrm{mL}}^{-1})}\times \;1000$$

Calibration curve for DPPH test is provided in Supplementary Figure S2.

## 3. Results and Discussion

### 3.1. Total Lipid Content, Fatty Acid Profile, and Lipid Quality Indicators of the Algae Blend

The total lipid content of the BLEND was estimated gravimetrically after the lipid extraction procedure as 4.02 ± 0.20 g 100 g^–1^ of DW biomass. The analysis of the fatty acid composition of the BLEND total lipid extract by GC-MS revealed the presence of 31 fatty acids (Table 1). The monounsaturated fatty acid (MUFA) 18:1 *n*-9 was detected as the most abundant fatty acid (30.10 ± 0.51%), followed by the saturated fatty acid (SFA) 16:0 (14.74 ± 0.31%) and the polyunsaturated fatty acids (PUFA) 18:3 *n*-3 (11.73 ± 0.33%) and 18:2 *n*-6 (11.58 ± 0.13%). The other identified fatty acids had relative abundances of less than 5%. The algae blend was characterized by containing a lower content of SFA (24.58 ± 0.63%), followed by MUFA (37.07 ± 0.74%) and PUFA (39.99 ± 0.29%). The total *n*-3 and *n*-6 fatty acid content reached, respectively, 19.42 ± 0.10% and 19.02 ± 0.32%.

The determined fatty acid profile of the BLEND has a particular composition when compared to those reported in the literature for the individual algae species that compose the mixture, i.e., *Chlorella vulgaris*, *Fucus vesiculosus*, and *Ulva rigida*. The FA 18:1 *n*-9 (oleic acid, OA) was described as the main fatty acid in *F. vesiculosus* (20–28%) [13,17,28], while in *U. rigida*, it can be presented only in relevant amounts (≈9%) [9,28]. The FA 16:0 was the most abundant SFA in all species [7,9,13,17,29,30], but some studies reported that it was the major fatty acid in *U. rigida*, reaching 42% [28,29,31]. Lastly, the essential fatty acids FA 18:3 *n*-3 (α-linolenic acid, ALA) and FA 18:2 *n*-6 (linoleic acid, LA) were identified at high levels in *C. vulgaris* (ALA: 24–26% and LA: 15–18%) [7,30], in contrast to the minor abundances reported for the seaweeds *F. vesiculosus* (ALA: 4–7% and LA: 7–9%) and *U. rigida* (ALA: 5–11% and LA: 1–2%).

Thus, in the BLEND, we were able to obtain a fatty acid profile characterized by a high content of OA, complemented by the presence of both essential fatty acids ALA and LA in interesting contents, therefore, a specific composition that was not achieved in the isolated algae species. OA, which is the main fatty acid in olive oil (>70% of the total fatty acids), has exhibited different health-promoting properties for the management and prevention of NCDs [32]. LA and ALA are essential fatty acids that need to be obtained through diet, as the human body is unable to synthesize them [33]. From LA and ALA, humans can synthesize, respectively, *n*-6 long chain PUFA, as 20:4 *n*-6 (arachidonic acid, AA), and *n*-3 long chain PUFA, as 20:5 *n*-3 (eicosapentaenoic acid, EPA) and 22:6 *n*-3 (docosahexaenoic acid, DHA) [33]. These fatty acids are valuable structural components of lipids in membranes and can have a substantial biological role because they are precursors of lipid mediators. Generally, AA is metabolized to lipid pro-inflammatory mediators, and EPA and DHA are precursors of anti-inflammatory signaling lipids, making these PUFA that play a crucial role in an extensive spectrum of biological processes, including inflammation and blood clotting [34].

To give new insight into the beneficial impact on health and assess the nutritional lipid quality of the BLEND, the PUFA/SFA and *n*-6/*n*-3 PUFA ratios, as well as the atherogenicity (AI) and thrombogenicity (TI) indices, were calculated (Table 2). The PUFA/SFA ratio was 1.63 ± 0.05 and the *n*-6/*n*-3 PUFA ratio was 0.98 ± 0.02, while the AI and TI were 0.41 ± 0.01 and 0.27 ± 0.01, respectively.

The ratio PUFA/SFA is the elementary index to estimate the impact of diet on cardiovascular health, and the higher this ratio, the more beneficial the effect [35]. The *n*-6/*n*-3 PUFA ratio is suggested as a relevant factor for brain development and decreasing the risk of developing non-communicable diseases, including autoimmune and neurodegenerative diseases [33]. The recommendations for healthy diets prioritize the ingestion of foods with *n*-6/*n*-3 PUFA ≤ 1; however, this recommended value is still controversial, and the absolute amounts of the fatty acids must be taken into account, too [33]. In addition, the indices AI and TI are the most commonly used theoretical calculations to measure the probability of reducing the risk of atherogenic plaques and blood clot formation, respectively [35]. The lower the AI and TI of a product, the higher the nutritional quality and the greater the potential to contribute to mitigating the prevalence of cardiovascular diseases. Our results are within the range described in the literature for the individual algae species (Table 2), other algae, fish, and shellfish [35]. Remarkably, some of the calculated values showed better results than those reported for seaweed blends [25]. Overall, the profitable values found support the nutritional potential of the BLEND total lipid extract, which could be useful to improve human diet quality and sustainability, and also to help prevent inflammatory, cardiovascular, and brain disorders.

### 3.2. Polar Lipidome of the Algae Blend

The polar lipidome of the BLEND total lipid extract was profiled by HILIC–ESI–HR-MS and MS/MS spectra analysis. Lipid species were identified based on the retention time information and accurate mass identification of ions detected in LC–MS data, and the comprehensive interpretation of MS/MS spectra allowed confirmation of the structural features of the polar head and fatty acid composition of lipid species, as described in detail by Rey et al. [27]. A total of 462 polar lipid species, distributed among glycolipids (GL, 118 species), phospholipids (PL, 206 species), and betaine lipids (BL, 138 species), were identified in the BLEND total lipid extract (Table 3).

Digalactosylmonoacylglycerol (DGMG), digalactosyldiacylglycerol (DGDG), diacylglyceryl-hydroxymethyl-N,N,N-trimethyl-β-alanine (DGTA), diacylglycerol-trimethyl homoserine (DGTS), lysophosphatidylcholine (LPC), lysophosphatidylethanolamine (LPE), lysophosphati-dylglycerol (LPG), lysophosphatidylinositol (LPI), monogalactosylmonoacylglycerol (MGMG), monogalactosyldiacylglycerol (MGDG), monoacylglyceryl-hydroxymethyl-N,N,N-trimethyl-β-alanine (MGTA), monoacylglycerol-trimethyl homoserine (MGTS), phosphatidic acid (PA), phosphatidylcholine (PC), phosphatidylethanolamine (PE), phosphatidylglycerol (PG), phosphatidylinositol (PI), phosphatidylserine (PS), sulfoquinovosyl diacylglycerol (SQDG), sulfoquinovosyl monoacylglycerol (SQMG).

**Figure 1:**
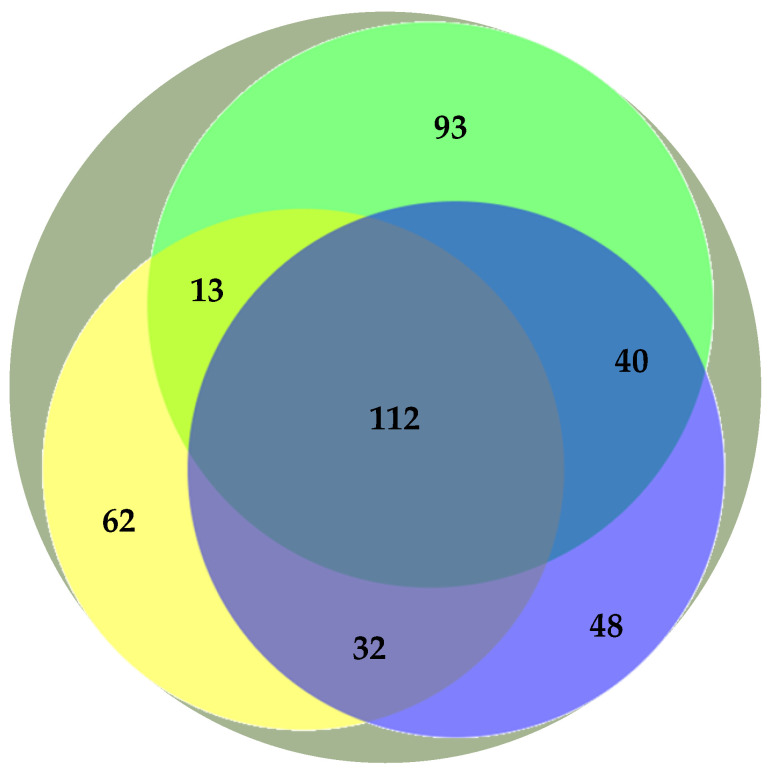
Venn diagram representation of the number of common polar lipid species identified in the algae blend (BLEND, 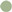) and reported in the literature for *Chlorella vulgaris* (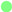), *Fucus vesiculosus* (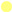), and *Ulva rigida* (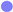).

Previous studies have addressed the polar lipidome of the individual algae species present in the BLEND (e.g., Refs. [13,15,29]). Actually, the BLEND lipid extract showed a greater diversity of lipid species in comparison with *C. vulgaris*, *F. vesiculosus*, and *U. rigida* (Table 3). To describe the relationship between the polar lipid profile of the BLEND and the three algae, the polar lipid species identified in the BLEND and reported until now for *C. vulgaris*, *F. vesiculosus*, and *U. rigida* were plotted on a Venn diagram (Figure 1). From the total of 462 lipid species identified in the BLEND lipid extract, only 112 were common to the three algae, while 93, 62, and 48 lipids were derived exclusively from *C. vulgaris*, *F. vesiculosus*, and *U. rigida*, respectively.

**Figure 2:**
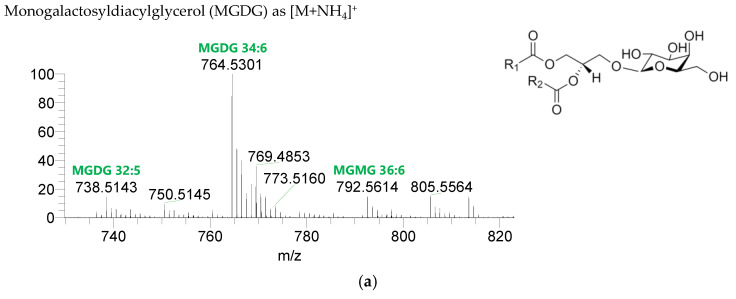

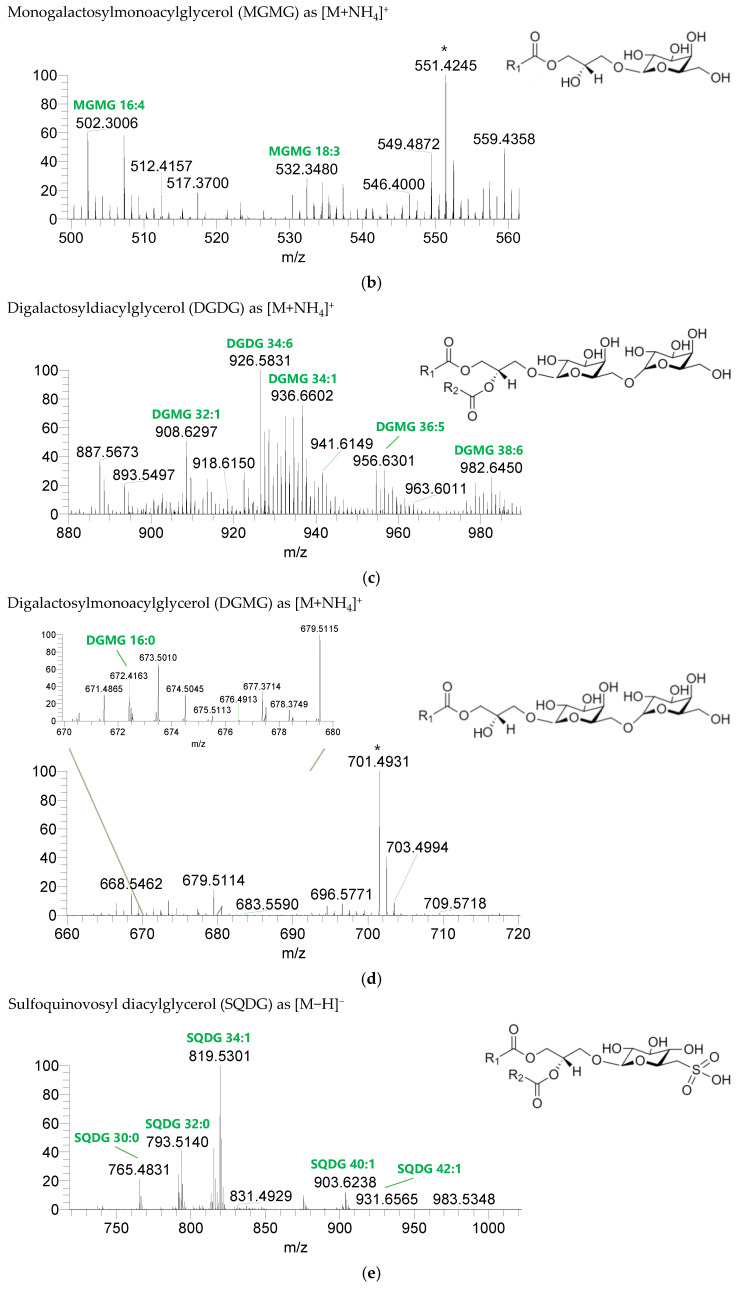

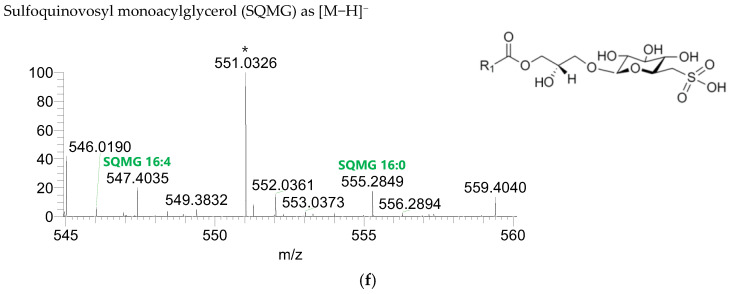
LC–MS spectra of glycolipid classes identified in the polar lipidome of the algae blend. (**a**) MGDG, (**b**) MGMG, (**c**) DGDG, and (**d**) DGMG were identified in the positive mode as [M+NH_4_]^+^ ions, and (**e**) SQDG and (**f**) SQMG were identified in the negative mode as [M−H]^−^ ions. The ions group assigned with symbol (asterisk) are a background.

The GL identified included four classes of galactolipids—monogalactosyldiacylglycerol (MGDG), monogalactosylmonoacylglycerol (MGMG), digalactosyldiacylglycerol (DGDG), and digalactosylmonoacylglycerol (DGMG)—identified as [M+NH_4_]^+^ ions (Table 4, Supplementary Table S1), and two classes of sulfolipids—sulfoquinovosyl diacylglycerol (SQDG) and sulfoquinovosyl monoacylglycerol (SQMG)—identified as [M−H]^−^ ions (Table 3) (Figure 2). A total of 29 lipid species of MGDG, 11 of MGMG, 33 of DGDG, 8 of DGMG, 34 of SQDG, and 3 of SQMG were recognized. The most abundant species in each class of glycolipids were: MGDG 34:6, assigned as MGDG (16:2_18:4), MGDG (16:3_18:3), and MGDG (16:4_18:2); MGMG 16:4; DGDG 34:6, assigned as DGDG (16:3_18:3); DGMG 16:0; SQDG 34:1, assigned as SQDG (16:0_18:1); and SQMG 16:0. GL species included mostly C16- and C18-fatty acyl chains, but also C14-, C15-, C17-, C19-, C22-, and C24-fatty acyl chains. 

**Figure 3:**
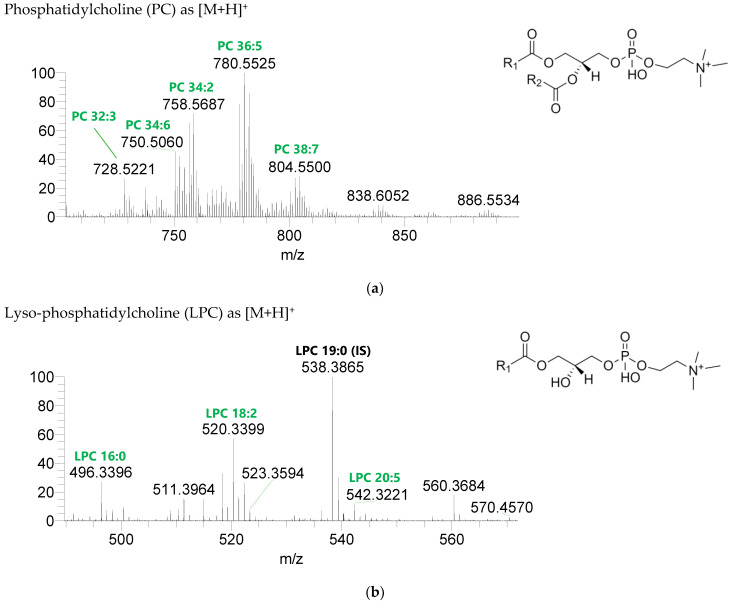

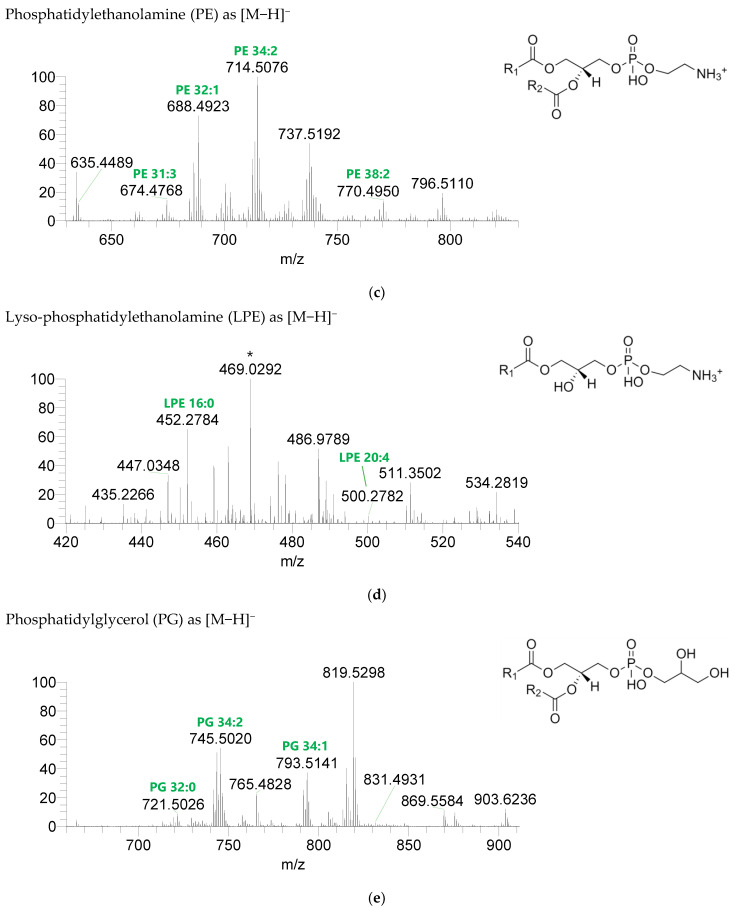

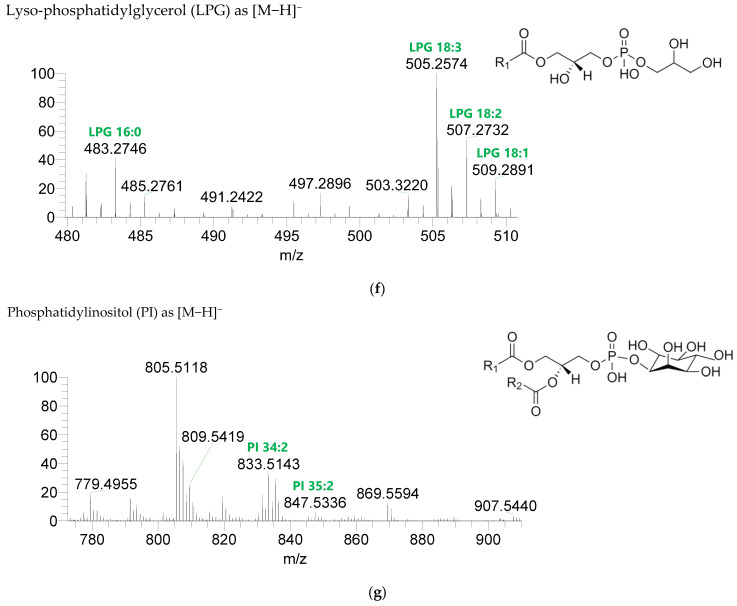
LC-MS spectra of phospholipids classes identified the polar lipidome of the algae blend. (**a**) PC and (**b**) LPC were identified in the positive mode as [M+H]^+^ ions, and (**c**) PE, (**d**) LPE, (**e**) PG, (**f**) LPG, and (**g**) PI were identified in the negative mode as [M-H]^-^ ions. The ions group assigned with symbol (asterisk) are a background.

Seven classes of PL were detected in the BLEND lipid extract: phosphatidylcholine (PC), lysophosphatidylcholine (LPC), phosphatidylethanolamine (PE), lysophosphatidylethanolamine (LPE), phosphatidylglycerol (PG), lysophosphatidylglycerol (LPG), and phosphatidylinositol (PI) (Figure 3, Table 5, Supplementary Table S2). PC and LPC were identified in the positive mode as [M+H]^+^ ions, and the remaining classes were identified in the negative mode as [M-H]^-^ ions. A total of 60 lipid species of PC, 21 of LPC, 47 of PE, 18 of LPE, 34 of PG, 5 of LPG, and 21 of PI were recognized. The most abundant species in each class of PL were: PC 36:5, assigned as PC (16:0_20:5), PC (16:1_20:4), and PC (18:2_18:3); LPC 18:2; PE 34:2, assigned as PE (16:0_18:2) and PE (16:1_18:1); LPE 16:0; PG 34:2, assigned as PG (16:0_18:2) and PG (16:1_18:1); LPG 18:3; and PI 34:2, assigned as PI (16:0_18:2) and PI (16:1_18:1). PL species were mainly esterified with C16-, C18-, and C20-fatty acyl chains, but also C14-, C15-, C17-, C19-, C21-, and C22-fatty acyl chains can be found esterified.

**Figure 4:**
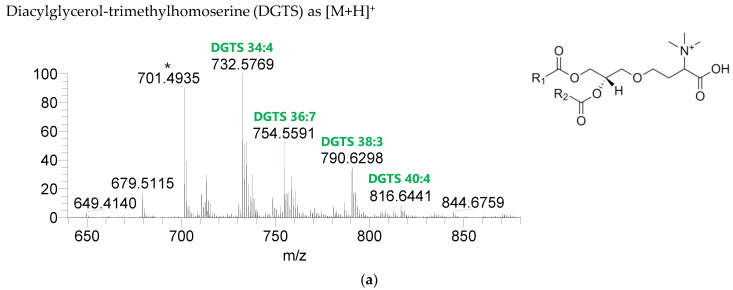

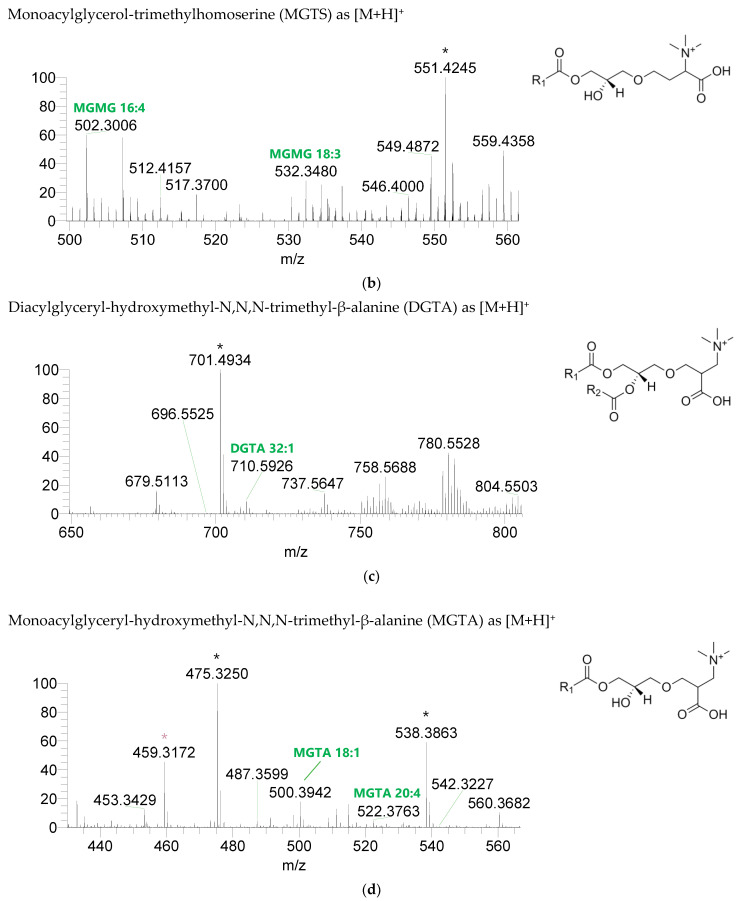
LC-MS spectra of betaine lipids classes identified the polar lipidome of the algae blend. (**a**) DGTS, (**b**) MGTS, (**c**) DGTA, and (**d**) MGTA were identified in the positive mode as [M+H]^+^ ions. The ions group assigned with symbol (asterisk) are a background.

The BL found in the BLEND lipid extract comprised four classes, including diacylglycerol-trimethyl homoserine (DGTS), monoacylglycerol-trimethyl homoserine (MGTS), diacylglyceryl-hydroxymethyl-N,N,N-trimethyl-β-alanine (DGTA), and monoacylglyceryl-hydroxymethyl-N,N,N-trimethyl-β-alanine (MGTA), which were identified in the positive mode as [M+H]^+^ ions (Figure 4, Table 6, Supplementary Table S3). A total of 61 lipid species of DGTS, 28 of MGTS, 36 of DGTA, and 13 of MGTA were identified. The most abundant species in each class of BL were: DGTS 34:4, assigned as DGTS (16:0_18:4); MGTS 16:0; DGTA 32:1, assigned as DGTS (14:0_18:1) and DGTS (16:0_16:1); and MGTA 18:1. Although C14-, C15-, C17-, C19-, C22-, and C24-fatty acyl chains can be detected esterified in BL species, they were primarily esterified with C16-, C18-, C20-, and C22-fatty acyl chains.

The GL species identified in the BLEND lipid extract were already described in *C. vulgaris*, *F. vesiculosus*, and/or *U. rigida* [13,29,30], with a few exceptions (Table 4). The PL fraction showed a vast diversity of phosphoglycerol-containing lipid species, mainly in comparison with the individual algae species (Table 3), with a strong contribution of lipid species from *C. vulgaris* (Table 5). However, we found a considerable number of PL species that were not described in lipidomic studies targeting *C. vulgaris*, *F. vesiculosus*, and *U. rigida* [9,13,15,16,29,30]. Finally, the arrangement of the three algae gave rise to a more complex BL profile, where DGTS and DGTA are present, as well as their lyso forms (Table 3). DGTS and MGTS are characteristically present in green seaweeds, such as *U. rigida* [16], and in microalgae only is the occurrence of DGTS more prominent [37]. The majority of the DGTS and MGTS species have been identified in at least one of the isolated algae species constituting the BLEND [9,12,13,14,16,29], mainly *U. rigida* (Table 6). The presence of DGTA and MGTA in the BLEND lipid extract is clearly derived from *F. vesiculosus* (Table 6), as these lipids are found most exclusively in Ochrophyta phylum [13,16]. Contrary to what was observed for the PL profile, the BL fraction benefits from the contribution of macroalgae rather than microalgae. Interestingly, BL are non-phosphorous zwitterionic polar glycerolipids analogous of PC and, together with PL, are considered the major polar lipids of cell membranes contributing to the maintenance of membrane architecture and functions due to the positively charged polar heads [38]. Thus, to conserve the charge and properties of membranes, in algae with lower levels of PL, BL are present in a higher amount (as noticed for *F. vesiculosus* and *U. rigida*), and vice-versa (as observed for *C. vulgaris*) [16,37]. Regarding the most abundant lipid species of each lipid class distinguished in the BLEND lipid extract, Table 3 shows that there is a correlation with what was reported for *C. vulgaris*, *F. vesiculosus*, and *U. rigida*.

In fact, some GL, PL, and BL species undetected in the individual algae were present in the BLEND. These lipid species were detected in very low abundances, which could be below the threshold used for lipid analysis in previous studies that identified the individual algae lipidomes. In addition, this could be related to changes in the lipid composition, namely, in terms of relative abundance, that may occur due to the adaptation of each algae to the growth conditions, as micro- and macroalgae samples analyzed in these other studies were not exactly the same ones used to formulate the blend sample under investigation here [13,30]. This incidence can be corroborated by other studies, as in the case of *U. rigida*, where minority species are not always likely to be identified [9,16,29]. Bear in mind that fluctuations in the lipidome can be observed in both wild and cultivated algae, in the latter to a lesser extent [16,29]. Another influencing factor was the approach to the analysis of the LC-MS data, because in the first studies that focused on the algae lipidome, which were carried out in our laboratory, lipid species with odd carbon numbers were not considered, such as MGDG 35:6, PC 31:4, and SQDG 31:1 [29,39]. Additionally, in the BLEND lipid extract, it was not possible to detect lipid species belonging to the PL classes phosphatidic acid (PA), phosphatidylserine (PS), and lysophosphatidylnositol (LPI). These classes have been identified before in *F. vesiculosus* (Table 3), but as lower abundant classes. The non-identification of these classes in the BLEND lipid extract could be due to a dilution effect or a suppression effect [27], which could mean that *F. vesiculosus* represents a minor percentage in the mixture.

It Is also important to point out that some polar lipid species found in *C. vulgaris*, *F. vesiculosus*, and/or *U. rigida* were not identified in the BLEND lipid extract. These lipid species were detected in very low abundance in the individual algae species [16]; therefore, in our study, signal suppression may have occurred, and the detection limit may not have been reached [27].

To sum up, the combination of *C. vulgaris*, *F. vesiculosus*, and *U. rigida* provides a single polar lipid profile, with a greater diversity of lipid species. The present identification of the polar lipids contained in the BLEND lipid extract is crucial to suit specific applications, as the numerous combinations of different acyl chains with the polar head give rise to GL, PL, and BL species with distinct properties and functions [26,37].

Marine GL have been exploited and characterized for being natural compounds with an extensive variety of molecular structures and biological activities, such as anti-inflammatory, antiproliferative, and antimicrobial [6]. The potential of the BLEND total lipid extract for biotechnological purposes is enhanced by the occurrence of several GL molecular species previously associated with bioactivity and potential health benefits of lipids. For instance, in the case of galactolipids, MGDG 34:4 (18:4_16:0) [40], MGDG 34:8 (18:4_16:4) [41], and MGMG 16:3 [42] displayed strong and dose-dependent nitric oxide inhibition, indicative of their potential as anti-inflammatory agents. MGDG 34:4 (18:4_16:0) and DGDG 34:4 (18:4_16:0) tested in two in vivo mouse models of inflammation showed anti-inflammatory activities and substantially lower toxicities than that of the reference treatments [43]. Antitumor properties have been unveiled in MGDG 32:4 (16:2/16:2), MGDG 32:5 (16:2_16:3), MGDG 34:4 (18:2_16:2), MGDG 34:6 (18:3_16:3), MGDG 36:4 (18:2/18:2), DGDG 34:4 (16:2_18:2), and DGDG 34:6 (16:3_18:3) from *C. vulgaris* [44], in MGDG 38:9 (18:4_20:5) from *Fucus evanescens* [45], and in MGDG 36:4 (20:4_16:0) from *Hydrolithon reinboldii* [46]. MGDG species with LA at the *sn*-2 position, as MGDG 32:2 (14:0/18:2) and MGDG 34:2 (16:0/18:2), have been suggested to play an important role in the inhibition of triglyceride accumulation in 3T3-L1 adipocytes [47]. Of particular note are the most abundant species of each sulfolipids class SQDG 34:1 (16:0_18:1) and SQMG 16:0, which have shown great antitumor activity against human carcinomas and antibacterial effect against *Bacillus subtilis* and *Escherichia coli* [48]. In addition, a study on *Chlorococcum* sp. suggested that SQDG bearing the bioactive fatty acid ALA or OA at the *sn*-2 position of their structure, as SQDG 34:1 (16:0/18:1) and SQDG 32:1 (14:0/18:1), are associated with strong anti-inflammatory and antithrombotic properties [20]. Meanwhile, SQDG 32:0 (16:0/16:0) isolated from a brown seaweed demonstrated potential as an antiviral agent against human herpesviruses [49]. The GL fraction of the BLEND appears to be a strong contender for a healthy diet, namely, as a more efficient bioavailable carrier of beneficial fatty acids, contributing to functional foods and ingredients, and a promising source of therapeutic agents intended for nutraceutical applications [6,19]. Alternatively, these bio-based and biodegradable food-grade compounds may be of interest as biosurfactants to prepare emulsions in food products or to develop drug delivery systems [50].

PL include a variety of versatile lipid species that are required in a wide range of biological processes. Studies on bioactivities of PL from algae are quite scarce [6]. Among the lipid species identified in the BLEND total lipid extract, LPC 16:0 and PG 34:2 (16:0_18:2) have already shown potential anti-inflammatory action [43,51]. Moreover, a body of evidence suggests that dietary marine PL have numerous health benefits, with a positive impact in several diseases—for example, age-related, cardiovascular, cognitive, and neurological disorders—especially PL esterified to *n*-3 PUFA [26,52]. The PL fraction of the BLEND is enriched in lipid species esterified to healthy fatty acids, such as 18:1, 18:2, and 18:3, and because marine PL products showed remarkably high stability against oxidation and better bioavailability [52,53], the application of this lipid fraction as ingredients for functional food, namely, food fortification, is promising [53]. Effectively, PL have been regularly and extensively used in a large set of industries. In short, the PL of the BLEND could be explored in the food (emulsifiers and fat-replacements), pharmaceutical (drug delivery systems), and cosmetic (emulsifiers and rehydrating agents for skincare) industries [26], as well as in neutron scattering research (mimics cell membranes) [54].

Although little is known about the BL bioactive potential, the molecular species MGTS 20:5 and DGTS 40:10 (20:5/20:5), detected in the BLEND lipid extract, were respectively described with antiatherogenic and anti-inflammatory activity [55,56]. More studies are needed to understand the bioactive potential of these abundant algae lipids.

### 3.3. Antioxidant Activity of the Algae Blend Total Lipid Extract

The antioxidant activity of the BLEND total lipid extract was evaluated using the ABTS^•+^ and DPPH^•^ scavenging assays (Table 7). The percentage of radical inhibition in the presence of the lipid extract was calculated after 120 min. An inhibition of 87.64 ± 2.29% of ABTS^•+^ and 37.98 ± 0.65% of DPPH^•^ was reached with the maximum tested concentration (250 μg mL^−1^). The concentration that provided 50% of ABTS^•+^ inhibition (IC_50_) was 140.01 ± 4.40 μg mL^−1^ with a TE of 126.56 ± 3.91 μmol Trolox g^−1^, while for DPPH^•^ assay, 35% of inhibition (IC_35_) was attained at a concentration of 230.62 ± 3.30 μg mL^−1^, representing a TE of 74.09 ± 1.07 μmol Trolox g^−1^.

Natural compounds with antioxidant activity are currently of interest mainly in the food and pharmaceutical industries [22]. Algae total lipid extracts contain lipids that have already been described as powerful antioxidants, such as GL, PL, pigments, and PUFA [15,57,58]. Therefore, recent studies have been focused on the antioxidant potential of total lipid extracts from different algae species [7,8,17,59,60]. The ABTS^•+^ and DPPH^•^ scavenging assays are chemical methods widely used to measure the antioxidant capacity of natural extracts, including algae and derived compounds, due to their low cost, operational simplicity, and radical stability, in spite of their biological irrelevance [60,61,62]. In our work, the BLEND lipid extract showed higher ability to scavenge the ABTS^•+^ than the DPPH^•^, as reported for natural extracts from fruits, vegetables, and algae [17,61], including *C. vulgaris*, *F. vesiculosus*, and *U. rigida* (Table 6). Effectively, our results are in agreement with those obtained for the individual algae species and with previous studies on other algae [7,59,60]. Overall, the BLEND total lipid extract showed antioxidant activity and can be used as a natural antioxidant, valued for promoting environmental and economic sustainability [2].

## 4. Conclusions

In this study, the fatty acid profile and the polar lipidome of an innovative blend of macro- and microalgae (BLEND) were determined for the first time. The essential fatty acids LA (18:2 *n*-6) and ALA (18:3 *n*-3) were among the most abundant fatty acids in the lipid pool, and the calculated lipid indicators included a well-balanced *n*-6/*n*-3 ratio and low AI and TI, which betoken a great nutritional value. Through a HILIC–ESI–MS/MS lipidomic approach, we identified 462 species of polar lipids, including glycolipids, phospholipids, and betaine lipids, some of which have already been recognized for their bioactive potential. The mixture of different macro- and microalgae species resulted in a blend characterized by a unique lipid profile, which could not be achieved by a single algae species. In addition, the BLEND total lipid extract displayed antioxidant activity. Ultimately, the mixture of different algae species can be an approach to achieve products that comprise more benefits privileging health, nutrition, and environmental sustainability. However, further studies are required. Nonetheless, this study acknowledges the BLEND as a promising sustainable source of natural bioactive lipid compounds with potential application, namely, in the food, nutraceutical, and cosmetic industries.

## Figures and Tables

**Table 1:** Fatty acid profile of the algae blend (BLEND) determined by GC-MS expressed in relative abundance (%). Values are expressed as the mean ± SD (*n* = 5).

Fatty Acids	Relative Abundance (%)
14:0	04.22 ± 0.32
16:0	14.74 ± 0.31
16:1 *n*-9	01.08 ± 0.06
16:1 *n*-7	01.76 ± 0.10
16:2 *n*-6	01.94 ± 0.03
16:3 *n*-3	04.06 ± 0.10
18:0	04.28 ± 0.79
18:1 *n*-9 (oleic acid, OA)	30.10 ± 0.51
18:1 *n*-7	02.29 ± 0.06
18:2 *n*-6 (linoleic acid, LA)	11.58 ± 0.13
18:3 *n*-4	01.15 ± 0.12
18:3 *n*-3 (α-linolenic acid, ALA)	11.73 ± 0.33
18:4 *n*-3	01.50 ± 0.10
20:4 *n*-6 (arachidonic acid, AA)	04.90 ± 0.19
20:5 *n*-3 (eicosapentaenoic acid, EPA)	01.53 ± 0.17
Others ^1^	04.73

^1^ Others = FAMEs with relative abundance < 1% (FA 14:1, 15:0, 16:1 *n*-5, 16:4 *n*-3, 17:0, 17:1, 17:2, 18:3 *n*-6, 19:1, 20:0, 20:1 *n*-9, 20:2 *n*-6, 20:3 *n*-6, 20:4 *n*-3, 22:0, 24:1 *n*-9).

**Table 2:** Lipid quality indicators values obtained for the algae blend (BLEND), considering the 31 fatty acids identified, in comparison with the calculated values reported in the literature for the algae species—*Chlorella vulgaris*, *Fucus vesiculosus*, and *Ulva rigida*.

Lipid Indicators	BLEND	*Chlorella vulgaris* [7,30]	*Fucus vesiculosus* [13,17,28]	*Ulva rigida* [9,12,17,28]
PUFA/SFA	1.63 ± 0.05	2.00–2.59	0.94–1.96	2.63
*n*-6/*n*-3 PUFA	0.98 ± 0.02	0.06–0.60	0.40–1.95	0.03–0.37
AI	0.41 ± 0.01	0.20–0.28	0.66–0.90	0.27–1.23
TI	0.27 ± 0.01	0.21	0.26–0.34	0.10–0.60

**Table 3:** Polar lipid profile of the BLEND total lipid extract identified by HILIC–ESI-MS/MS with the indication of the number of lipid species identified and the most abundant lipid species by the class of polar lipids, in comparison with polar lipid profiles of the individual algae species—*Chlorella vulgaris*, *Fucus vesiculosus*, and *Ulva rigida*—from literature. The number of lipid species is related to the molecular weight determined by MS and corresponds to the number of identified lipid molecular ions.

Polar Lipids Classes	BLEND		*Chlorella vulgaris* [14,15,30,36]		*Fucus vesiculosus* [13,16]		*Ulva rigida* [9,12,16,29]	
	Nº	Major Lipid Species	Nº	Major Lipid Species	Nº	Major Lipid Species	Nº	Major Lipid Species
**Glycolipids (GL)**								
Total GL		118		66		93		103
MGDG	29	34:6	17	34:6	23	38:7 or 38:8	29	34:8
MGMG	11	16:4	5	18:3	9	16:4 or 18:4	13	16:4
DGDG	33	34:6	23	34:6	23	38:8	21	34:1 or 34:3
DGMG	8	16:0	3	16:0	5	18:3	10	16:0 or 16:4
SQDG	34	34:1	18	32:0	26	34:1	25	34:1
SQMG	3	16:0	0	–	7	16:0	5	16:0
**Phospholipids (PL)**								
Total PL		206		160		82		93
PA	0	–	0	–	3	40:8	0	–
PC	60	36:5	54	36:6	19	36:2 or 36:3 or 38:7	24	36:2
LPC	21	18:2	16	18:2	6	18:1	9	16:0
PE	47	34:2	41	34:2	19	40:8	21	30:3 or 32:1 or 34:2
LPE	18	16:0	13	16:1 or 18:1	6	20:4	7	16:1 or 22:5
PG	34	34:2	23	34:3	11	34:4	18	34:4
LPG	5	18:3	2	16:0	3	16:1	4	16:1
PI	21	34:2	10	34:2	12	34:1 or 34:2	9	34:1 or 34:2 or 34:3
LPI	0	–	0	–	2	18:1	1	16:0
PS	0	–	0	–	1	30:3	0	–
**Betainelipids (BL)**								
Total BL		138		43		81		68
DGTS	61	34:4	29	34:4	17	32:1	47	32:1 or 34:4
MGTS	28	16:0	14	nd	8	16:0 or 18:3	21	16:0 or 18:4
DGTA	36	32:1	0	–	43	32:1	0	–
MGTA	13	18:1	0	–	13	18:1	0	–
Total	454		269		256		264	

nd: not defined.

**Table 4:** Glycolipids identified in the polar lipidome of the algae blend (BLEND) by high resolution HILIC–ESI–MS and MS/MS. C carbons, N number of double bonds. * Lipid species identified based on the polar head fragment, calculated mass, and retention time. ** Lipid species identified based on calculated mass and retention time. The presence of the lipid species in the individual algae species—*Chlorella vulgaris*, *Fucus vesiculosus*, and *Ulva rigida*—is indicated by a cross (×), according to the reports in the literature.

Lipid Species (C:N)	Observed *m/z*	Fatty Acyl Chains (C:N)	*Chlorella vulgaris* [14,15,30,36]	*Fucus* *Vesiculosus* [13,16]	*Ulva rigida* [9,12,16,29]
**MGDG identified as [M+NH_4_]^+^**					
MGDG 30:4	712.4980	**			×
MGDG 32:1	746.5765	**		×	
MGDG 32:2	744.5604	**	×	×	
MGDG 32:3	742.5447	**	×	×	×
MGDG 32:4	740.5289	**	×	×	×
MGDG 32:5	738.5144	14:1_18:4 and 16:1_16:4 and 16:2_16:3	×		×
MGDG 32:6	736.4986	16:2_16:4 and 16:3/16:3	×		×
MGDG 34:1	774.6058	**	×	×	×
MGDG 34:2	772.5925	**	×	×	
MGDG 34:3	770.5747	16:0_18:3 and 16:3_18:0	×	×	×
MGDG 34:4	768.5607	16:0_18:4 and 16:1_18:3 and 16:2_18:2	×	×	×
MGDG 34:5	766.5441	**	×		×
MGDG 34:6	764.5302	16:2_18:4 and 16:3_18:3 and 16:4_18:2	×	×	×
MGDG 34:8	760.4984	16:4_18:4			×
MGDG 34:9	758.4871	*			×
MGDG 35:1	784.5930	**	×		
MGDG 35:6	778.5442	17:3_18:3			
MGDG 36:4	796.5921	18:1_18:3 and 18:2_18:2	×	×	×
MGDG 36:5	794.5766	**	×	×	
MGDG 36:6	792.5615	18:3/18:3	×	×	×
MGDG 36:7	790.5487	**		×	×
MGDG 36:8	788.5333	**		×	×
MGDG 36:9	786.5195	**		×	×
MGDG 38:5	822.6093	**		×	×
MGDG 38:6	820.5954	18:2_20:4		×	
MGDG 38:7	818.5770	**		×	×
MGDG 38:8	816.5594	**		×	
MGDG 38:9	814.5447	**		×	
MGDG 40:9	842.5744	**		×	
		**MGMG identified as [M+NH_4_]^+^**			
MGMG 16:0	510.3633	**	×	×	×
MGMG 16:1	508.3484	**		×	×
MGMG 16:2	506.3328	**	×		×
MGMG 16:3	504.3172	**	×	×	×
MGMG 16:4	502.3006	**		×	×
MGMG 18:1	536.3795	**		×	×
MGMG 18:2	534.3642	**	×	×	×
MGMG 18:3	532.3479	**	×	×	×
MGMG 18:4	530.3320	**		×	×
MGMG 20:4	558.3630	**			×
MGMG 20:5	556.3481	**		×	×
**DGDG identified as [M+NH_4_]^+^**					
DGDG 28:0	854.5833	*	×		
DGDG 30:0	882.6153	*			×
DGDG 32:0	910.6476	**	×		×
DGDG 32:1	908.6297	14:0_18:1	×	×	×
DGDG 32:2	906.6156	14:0_18:2	×	×	
DGDG 32:3	904.6006	14:0_18:3 and 16:0_16:3	×	×	
DGDG 32:4	902.5836	14:0_18:4 and 16:1_16:3 and 16:2/16:2	×		×
DGDG 32:5	900.5676	16:1_16:4 and 16:2_16:3	×		
DGDG 34:1	936.6602	16:0_18:1 and 16:1_18:0	×	×	×
DGDG 34:2	934.6447	16:0_18:2 and 16:1_18:1 and 17:0_17:2	×	×	×
DGDG 34:3	932.6294	16:0_18:3 and 16:1_18:2 and 16:2_18:1	×	×	×
DGDG 34:4	930.6140	16:0_18:4 and 16:1_18:3 and 16:2_18:2 and 16:3_18:1	×	×	×
DGDG 34:5	928.5969	16:2_18:3 and 16:3_18:2	×	×	×
DGDG 34:6	926.5831	16:3_18:3	×		×
DGDG 34:7	924.5653	**	×		×
DGDG 34:8	922.5515	16:4_18:4			×
DGDG 35:1	950.6733	**	×		
DGDG 35:2	948.6634	**	×		
DGDG 35:3	946.6464	17:0_18:3	×		
DGDG 36:1	964.6890	*		×	
DGDG 36:2	962.6748	**	×	×	×
DGDG 36:3	960.6590	**	×	×	
DGDG 36:4	958.6438	**	×	×	×
DGDG 36:5	956.6301	**	×	×	×
DGDG 36:6	954.6157	18:3/18:3	×	×	×
DGDG 36:7	952.6031	**	×	×	×
DGDG 36:8	950.5872	**		×	
DGDG 36:9	948.5730	**			
DGDG 38:5	984.6586	**		×	×
DGDG 38:6	982.6450	18:1_20:5		×	×
DGDG 38:7	980.6286	**		×	×
DGDG 38:8	978.6140	18:3_20:5		×	
DGDG 38:9	976.6000	**		×	
**DGMG identified as [M+NH_4_]^+^**					
DGMG 16:0	672.4163	**	×	×	×
DGMG 16:1	670.4004	**			×
DGMG 16:4	664.3538	**			×
DGMG 18:1	698.4322	**		×	
DGMG 18:2	696.4172	*	×	×	
DGMG 18:3	694.4015	**	×	×	×
DGMG 18:4	692.3839	**		×	×
DGMG 20:5	718.4000	**			×
**SQDG identified as [M+H]^–^**					
SQDG 28:0	737.4519	14:0/14:0		×	×
SQDG 30:0	765.4831	14:0_16:0	×	×	×
SQDG 30:1	763.4671	*		×	×
SQDG 30:4	757.4215	14:0_16:4			
SQDG 31:1	777.4821	15:0_16:1			
SQDG 32:0	793.5142	16:0/16:0	×	×	×
SQDG 32:1	791.4987	14:0_18:1	×	×	×
SQDG 32:2	789.4831	14:0_18:2 and 16:0_16:2	×	×	×
SQDG 32:3	787.4676	14:0_18:3 and 16:0_16:3 and 16:1_16:2	×	×	×
SQDG 32:4	785.4528	**	×		×
SQDG 33:0	807.5259	**			
SQDG 33:1	805.5113	14:0_19:1 and 15:0_18:1 and 16:1_17:0			×
SQDG 33:3	801.4830	15:0_18:3			
SQDG 34:0	821.5433	14:0_20:0 and 16:0_18:0	×		
SQDG 34:1	819.5302	16:0_18:1	×	×	×
SQDG 34:2	817.5118	*	×	×	×
SQDG 34:3	815.4987	16:0_18:3	×	×	×
SQDG 34:4	813.4834	16:0_18:4	×	×	×
SQDG 34:5	811.4678	16:2_18:3	×		×
SQDG 34:6	809.4519	16:4_18:2			
SQDG 36:0	849.5737	*		×	
SQDG 36:1	847.5602	18:0_18:1	×	×	×
SQDG 36:2	845.5452	**	×	×	×
SQDG 36:3	843.5280	**	×	×	×
SQDG 36:4	841.5137	18:2/18:2	×	×	×
SQDG 36:5	839.4970	*	×	×	×
SQDG 36:6	837.4830	18:3/18:3	×	×	×
SQDG 38:0	877.6068	**		×	×
SQDG 38:1	875.5922	14:0_24:1 and 16:0_22:1		×	
SQDG 38:5	867.5301	**		×	×
SQDG 40:1	903.6240	16:0_24:1		×	
SQDG 42:1	931.6566	**		×	
SQDG 42:8	917.5426	**			
SQDG 44:5	951.6221	**			
**SQMG identified as [M+H]^–^**					
SQMG 16:0	555.2839	**		×	×
SQMG 16:4	547.2213	**			
SQMG 18:1	581.2996	18:1		×	×

**Table 5:** Phospholipids identified in the polar lipidome of the algae blend (BLEND) by high resolution HILIC-ESI-MS and MS/MS. C carbons, N number of double bonds. * Lipid species identified based on the polar head fragment, calculated mass, and retention time. ** Lipid species identified based on calculated mass and retention time. The presence of the lipid species in the individual algae species—*Chlorella vulgaris*, *Fucus vesiculosus*, and *Ulva rigida*—is indicated by a cross (×), according to the reports in the literature.

Lipid Species (C:N)	Calculated *m/z*	Fatty Acyl Chains (C:N)	*Chlorella vulgaris* [14,15,30,36]	*Fucus vesiculosus* [13,16]	*Ulva rigida* [9,12,16,29]
		**PC identified as [M+H]^+^**			
PC 30:0	706.5389	**	×		×
PC 30:3	700.4890	**	×		
PC 31:1	718.5362	15:0_16:1 and 15:1_16:0	×		
PC 31:3	714.5067	15:0_16:3 and 15:1_16:2	×		
PC 31:4	712.4907	**			
PC 32:0	734.5679	**	×		
PC 32:1	732.5521	16:0_16:1	×		×
PC 32:2	730.5370	16:0_16:2 and 16:1_16:1 and 14:0_18:2	×	×	×
PC 32:3	728.5224	16:0_16:3 and 16:1_16:2	×		
PC 32:4	726.5065	16:1_16: and 16:2/16:2	×		
PC 32:5	724.4914	16:2_16:3	×		
PC 32:6	722.4753	16:3/16:3	×		
PC 33:2	744.5528	**	×		
PC 33:3	742.5381	15:0_18:3 and 16:1_17:2 and 16:2_17:1 and 16:3_17:0	×		
PC 33:4	740.5223	16:2_17:2 and 16:3_17:1 and 16:4_17:0	×		
PC 33:5	738.5068	**	×		
PC 33:6	736.4907	16:3_17:3			
PC 34:1	760.5820	16:0_18:1 and 16:1_18:0	×	×	×
PC 34:2	758.5688	*	×	×	×
PC 34:3	756.5535	16:0_18:3 and 16:1_18:2 and 16:2_18:1 and 16:3_18:0	×	×	×
PC 34:4	754.5374	16:0_18:4 and 16:1_18:3 and 16:2_18:2 and 16:3_18:1 and 16:4_18:0	×		×
PC 34:5	752.5216	16:1_18:4 and 16:2_18:3 and 16:3_18:2	×		
PC 34:6	750.5063	16:3_18:3	×		
PC 34:7	748.4904	**	×		
PC 34:8	746.4734	**	×		
PC 34:9	744.4590	*	×		
PC 35:1	774.5998	17:0_18:1	×		
PC 35:2	772.5842	17:0_18:2	×		
PC 35:3	770.5689	17:0_18:3 and 17:1_18:2 and 17:2_18:3	×		
PC 35:4	768.5526	17:1_18:3 and 17:2_18:2	×		
PC 35:5	766.5367	17:2_18:3 and 17:3_18:2	×		
PC 35:6	764.5212	**	×		
PC 35:7	762.5049	*	×		
PC 35:9	758.4750	*			
PC 36:2	786.6020	16:1_20:1 and 17:1_19:1 and 18:0_18:2 and 18:1/18:1	×	×	×
PC 36:3	784.5842	18:0_18:3 and 18:1_18:2	×	×	×
PC 36:4	782.5690	16:1_20:3 and 16:2_20:2 and 18:1_18:3 and 18:2/18:2	×		×
PC 36:5	780.5535	16:0_20:5 and 16:1_20:4 and 18:2_18:3	×	×	×
PC 36:6	778.5372	16:3_20:3 and 18:2_18:4 and 18:3/18:3	×	×	×
PC 36:7	776.5212	**	×	×	×
PC 36:8	774.5038	**	×	×	×
PC 36:9	772.4887	**	×		
PC 37:3	798.6003	**	×		
PC 37:4	796.5851	18:3_19:1	×		
PC 38:2	814.6302	18:1_20:1 and 18:2_20:0	×		×
PC 38:3	812.6163	18:1_20:2 and 18:2_20:1 and 18:3_20:0	×		×
PC 38:4	810.6008	*	×		
PC 38:5	808.5827	18:2_20:3 and 18:3_20:2	×	×	×
PC 38:6	806.5663	18:2_20:4 and 18:3_20:3	×	×	×
PC 38:7	804.5525	**	×	×	×
PC 38:8	802.5367	**	×	×	×
PC 38:9	800.5199	**	×	×	×
PC 39:9	814.5347	**			
PC 40:6	834.5972	**	×		
PC 40:7	832.5833	**		×	
PC 40:8	830.5668	**		×	×
PC 40:9	828.5521	**		×	×
PC 40:10	826.5352	**		×	×
PC 44:2	898.7245	**			
PC 44:3	896.7108	**			
		**LPC identified as [M+H]^+^**			
LPC 14:0	468.3085	**			×
LPC 16:0	496.3395	16:0	×	×	×
LPC 16:1	494.3241	16:1	×	×	×
LPC 16:2	492.3083	16:2	×		
LPC 16:3	490.2925	16:3	×		
LPC 17:0	510.3551	17:0	×		
LPC 17:1	508.3391	17:1	×		
LPC 17:3	504.3075	17:3			
LPC 18:0	524.3709	18:0	×		
LPC 18:1	522.3563	**	×	×	×
LPC 18:2	520.3400	18:2	×		
LPC 18:3	518.3237	18:3	×	×	×
LPC 19:1	536.3723	19:1			
LPC 19:2	534.3546	19:2			
LPC 20:1	550.3869	20:1	×		
LPC 20:2	548.3696	20:2			
LPC 20:3	546.3536	**	×		
LPC 20:4	544.3387	**	×		×
LPC 20:5	542.3235	20:5	×	×	×
LPC 22:5	570.3542	**	×		
LPC 22:6	568.3385	**	×	×	×
		**PE identified as [M-H]^–^**			
PE 28:1	632.4302	14:0_14:1			
PE 29:0	648.4611	14:0_15:0			
PE 29:1	646.4456	14:1_15:0	×		
PE 30:0	662.4770	15:0/15:0	×	×	×
PE 30:1	660.4612	14:0_16:1 and 14:1_16:0	×	×	
PE 30:2	658.4467	14:0_16:2	×		
PE 30:3	656.4320	**	×		×
PE 31:1	674.4766	15:0_16:1	×		
PE 31:2	672.4615	15:0_16:2	×		
PE 31:3	670.4462	15:0_16:3	×		
PE 32:1	688.4922	16:0_16:1	×		×
PE 32:2	686.4768	16:0_16:2 and 16:1/16:1	×	×	×
PE 32:3	684.4612	14:0_18:3 and 16:0_16:3 and 16:1_16:2	×		
PE 32:4	682.4463	16:1_16:3 and 16:2/16:2	×		×
PE 32:5	680.4296	**	×		
PE 32:6	678.4140	*	×		
PE 33:1	702.5079	**	×		
PE 33:2	700.4920	15:0_18:2 and 16:1_17:1			
PE 33:3	698.4770	15:0_18:3 and 16:3_17:0	×		
PE 34:1	716.5203	16:0_18:1	×	×	×
PE 34:2	714.5075	16:0_18:2 and 16:1_18:1	×	×	×
PE 34:3	712.4924	16:0_18:3 and 16:1_18:2 and 16:2_18:1	×	×	×
PE 34:4	710.4767	16:1_18:3 and 16:2_18:2 and 16:3_18:1	×	×	
PE 34:5	708.4613	16:2_18:3 and 16:3_18:2	×	×	×
PE 34:6	706.4456	16:3_18:3	×		
PE 35:2	728.5236	17:0_18:2 and 17:1_18:1	×		
PE 35:3	726.5079	17:0_18:3 and 17:1_18:2 and 17:2_18:1	×		
PE 35:4	724.4924	17:0_18:4 and 17:1_18:3 and 17:2_18:2	×		
PE 35:5	722.4765	17:2_18:3	×		
PE 35:6	720.4610	17:3/18:3	×		
PE 36:2	742.5387	18:0_18:2 and 18:1/18:1	×		×
PE 36:3	740.5227	**	×	×	×
PE 36:4	738.5090	18:1_18:3 and 18:2_18:2	×	×	×
PE 36:5	736.4923	*	×	×	×
PE 36:6	734.4769	18:3/18:3	×		×
PE 37:3	754.5374	18:2_19:1	×		
PE 37:4	752.5234	18:3_19:1	×		
PE 37:5	750.5075	18:3_19:2	×		
PE 38:2	770.5696	18:1_20:1 and 18:2_20:0			
PE 38:3	768.5545	18:1_20:2 and 18:2_20:1 and 18:3_20:0			
PE 38:4	766.5389	18:2_20:2 and 18:3_20:1			
PE 38:5	764.5219	*			
PE 38:7	760.4909	*	×	×	
PE 40:8	786.5082	20:4/20:4		×	×
PE 40:9	784.4915	*		×	×
PE 40:11	780.4598	*			
PE 42:2	826.6337	**			
**LPE identified as [M-H]^–^**					
LPE 14:0	424.2469	*	×		
LPE 15:0	438.2626	*	×		
LPE 16:0	452.2783	*	×		×
LPE 16:1	450.2626	16:1	×	×	×
LPE 16:2	448.2470	**	×		
LPE 16:3	446.2314	**	×		
LPE 17:0	466.2939	*	×		
LPE 17:1	464.2780	17:1	×		
LPE 18:1	478.2940	18:1	×	×	×
LPE 18:2	476.2782	18:2	×	×	×
LPE 18:3	474.2624	18:3	×	×	
LPE 19:1	492.3097	19:1			
LPE 20:0	508.3410	**			
LPE 20:1	506.3255	**			
LPE 20:4	500.2784	20:4	×	×	×
LPE 20:5	498.2626	**	×	×	×
LPE 22:1	534.3576	*			
LPE 22:5	526.2938	*			×
**PG identified as [M-H]^–^**					
PG 28:1	663.4221	14:0_14:1			
PG 30:0	693.4711	14:0_16:0	×		×
PG 30:1	691.4542	**	×		×
PG 32:0	721.5026	16:0/16:0	×	×	×
PG 32:1	719.4870	16:0_16:1	×	×	×
PG 32:2	717.4714	16:0_16:2 and 16:1/16:1	×	×	×
PG 32:3	715.4552	*			
PG 32:4	713.4409	14:0_18:3 and 16:1_16:3 and 16:2/16:2			×
PG 32:5	711.4210	**			×
PG 33:0	735.5186	**	×		
PG 33:1	733.5025	15:0_18:1 and 16:0_17:1 and 16:1_17:0	×		
PG 33:2	731.4868	15:0_18:2 and 16:0_17:2 and 16:1_17:1	×		
PG 33:3	729.4714	15:0_18:3 and 16:3_17:0	×		
PG 34:0	749.5309	14:0_20:0 and 16:0_18:0	×	×	×
PG 34:1	747.5154	16:0_18:1 and 16:1_18:0	×	×	×
PG 34:2	745.5020	16:0_18:2 and 16:1_18:1	×	×	×
PG 34:3	743.4869	16:0_18:3 and 16:1_18:2	×	×	×
PG 34:4	741.4714	14:0_20:4 and 16:0_18:4 and 16:1_18:3 and 16:2_18:2	×	×	×
PG 34:5	739.4559	14:0_20:5 and 16:1_18:4 and 16:2_18:3 and 16:3_18:2 and 16:4_18:1	×		×
PG 35:2	759.5170	14:0_21:2 and 17:0_18:2	×		
PG 35:3	757.5018	14:0_21:3 and 17:0_18:3	×		
PG 35:4	755.4859	17:1_18:3	×		
PG 35:6	751.4556	17:2_18:4			
PG 36:1	775.5458	**			
PG 36:2	773.5334	18:0_18:2 and 18:1/18:1	×	×	×
PG 36:3	771.5174	**	×	×	×
PG 36:4	769.5020	16:0_20:4 and 16:1_20:3 and 18:1_18:3 and 18:2/18:2	×	×	×
PG 36:5	767.4860	**	×		×
PG 36:9	759.4227	*			
PG 38:5	795.5207	18:1_20:4 and 18:2_20:3 and 18:3_20:2	×		
PG 40:11	811.4561	*			
PG 42:11	839.4862	*			
PG 44:5	879.6088	**			
PG 46:5	907.6429	**			
**LPG identified as [M–H]^–^**					
LPG 16:0	483.2744	16:0	×	×	×
LPG 16:1	481.2571	*		×	×
LPG 18:1	509.2893	**		×	×
LPG 18:2	507.2732	**			
LPG 18:3	505.2573	18:3	×		×
**PI identified as [M–H]^–^**					
PI 30:0	781.4844	**			×
PI 30:2	777.4545	14:0_16:2 and 14:1_16:1			
PI 32:3	803.4719	**	×		
PI 34:0	837.5458	**		×	
PI 34:1	835.5308	16:0_18:1	×	×	×
PI 34:2	833.5185	16:0_18:2 and 16:1_18:1	×	×	×
PI 34:3	831.5037	16:0_18:3 and 16:1_18:2	×	×	×
PI 34:4	829.4841	16:0_18:4 and 16:1_18:3 and 16:2_18:2			×
PI 34:5	827.4712	14:0_20:5 and 16:1_18:4 and 16:2_18:3 and 16:3_18:2 and 16:4_18:1	×		
PI 35:2	847.5334	15:0_20:2 and 17:0_18:2	×		
PI 36:1	863.5620	**		×	
PI 36:2	861.5478	**	×	×	
PI 36:3	859.5336	16:0_20:3 and 16:1_20:2 and 16:2_20:1 and 18:0_18:3 and 18:2_18:1	×	×	
PI 36:4	857.5166	16:0_20:4 and 18:0_18:4 and 18:1_18:3 and 18:2/18:2	×	×	×
PI 36:5	855.5034	16:0_20:5 and 18:1_18:4 and 18:2_18:3	×		
PI 36:6	853.4895	**			
PI 38:4	885.5527	18:0_20:4 and 18:1_20:3 and 18:2_20:2 and 18:3_20:1			×
PI 38:5	883.5373	**			
PI 38:8	877.4843	**		×	×
PI 40:7	907.5305	**		×	
PI 40:10	901.4855	**			

**Table 6:** Betaine lipids identified in the polar lipidome of the algae blend (BLEND) by high resolution HILIC–ESI–MS and MS/MS. C carbons, N number of double bonds. * Lipid species identified based on the polar head fragment, calculated mass, and retention time. ** Lipid species identified based on calculated mass and retention time. The presence of the lipid species in the individual algae species—*Chlorella vulgaris*, *Fucus vesiculosus*, and *Ulva rigida*—is indicated by a cross (×), according to the reports in the literature.

Lipid Species (C:N)	Calculated *m/z*	Fatty Acyl Chains (C:N)	*Chlorella vulgaris* [14,15,30,36]	*Fucus vesiculosus* [13,16]	*Ulva rigida* [9,12,16,29]
**DGTS identified as [M+H]^+^**					
DGTS 28:0	656.5463	*		×	×
DGTS 30:0	684.5781	14:0_16:0	×	×	×
DGTS 30:1	682.5614	14:0_16:1	×	×	×
DGTS 30:3	678.5291	14:0_16:3	×		×
DGTS 30:4	676.5144	**			×
DGTS 32:0	712.6080	16:0/16:0	×		×
DGTS 32:1	710.5924	14:0_18:1 and 16:0_16:1	×	×	×
DGTS 32:2	708.5768	14:0_18:2 and 16:0_16:2 and 16:1/16:1		×	×
DGTS 32:3	706.5599	*	×	×	×
DGTS 32:4	704.5455	14:0_18:4 and 16:0_16:4		×	×
DGTS 32:6	700.5140	**			×
DGTS 33:1	724.6093	16:0_17:1	×		
DGTS 33:2	722.5925	16:0_17:2	×		
DGTS 33:3	720.5790	15:0_18:3	×		
DGTS 33:4	718.5612	15:0_18:4	×		
DGTS 34:1	738.6232	16:0_18:1	×	×	×
DGTS 34:2	736.6066	16:0_18:2 and 16:1_18:1	×	×	×
DGTS 34:3	734.5915	16:0_18:3	×	×	×
DGTS 34:4	732.5768	16:0_18:4	×	×	×
DGTS 34:5	730.5608	16:1_18:4 and 16:2_18:3	×		×
DGTS 34:6	728.5463	*	×		×
DGTS 34:7	726.5286	16:4_18:3	×		×
DGTS 34:8	724.5159	16:4_18:4			×
DGTS 35:4	746.5931	15:0_20:4 and 17:1_18:3	×		
DGTS 35:5	744.5763	17:1_18:4	×		
DGTS 36:1	766.6528	16:0_20:1 and 18:0_18:1			
DGTS 36:2	764.6399	16:0_20:2 and 18:1/18:1	×	×	×
DGTS 36:3	762.6231	16:0_20:3	×	×	
DGTS 36:4	760.6069	16:0_20:4 and 18:1_18:3	×		×
DGTS 36:5	758.5923	16:0_20:5 and 18:1_18:4 and 18:2_18:3	×	×	×
DGTS 36:6	756.5743	18:3/18:3	×	×	×
DGTS 36:7	754.5594	18:3_18:4	×	×	×
DGTS 36:8	752.5452	18:4/18:4		×	×
DGTS 36:9	750.5328	**			×
DGTS 36:10	748.5142	*			
DGTS 38:0	796.7023	**			×
DGTS 38:1	794.6881	18:1_20:0			
DGTS 38:3	790.6542	*			
DGTS 38:5	786.6238	18:0_20:5	×		×
DGTS 38:6	784.6072	16:0_22:5	×		×
DGTS 38:7	782.5901	**			×
DGTS 38:8	780.5744	18:4_20:4			×
DGTS 38:9	778.5592	18:4_20:5			×
DGTS 38:10	776.5439	*			×
DGTS 40:3	818.6850	**			×
DGTS 40:4	816.6710	16:0_24:4 and 18:4_22:0			×
DGTS 40:5	814.6525	**			×
DGTS 40:6	812.6402	18:1_22:5			×
DGTS 40:7	810.6229	**			×
DGTS 40:8	808.6066	18:3_22:5			×
DGTS 40:9	806.5920	18:4_22:5	×		×
DGTS 40:10	804.5748	20:5/20:5	×		
DGTS 42:5	842.6879	**			×
DGTS 42:6	840.6688	**			×
DGTS 42:7	838.6531	20:3_22:4			
DGTS 42:9	834.6230	**			
DGTS 42:10	832.6071	**			×
DGTS 42:11	830.5905	**			×
DGTS 44:5	870.7184	22:0_22:5			×
DGTS 44:10	860.6385	22:5/22:5			×
DGTS 44:12	856.6055	**			
**MGTS identified as [M+H]^+^**					
MGTS 14:0	446.3472	14:0	×	×	×
MGTS 15:0	460.3635	*	×		
MGTS 16:0	474.3788	*	×	×	×
MGTS 16:1	472.3633	*	×	×	×
MGTS 16:2	470.3478	**			×
MGTS 16:3	468.3343	*	×		×
MGTS 16:4	466.3162	*			×
MGTS 17:1	486.3787	*	×		
MGTS 17:3	482.3462	*			
MGTS 18:0	502.4091	*	×		×
MGTS 18:1	500.3941	*	×	×	×
MGTS 18:2	498.3800	*	×	×	×
MGTS 18:3	496.3613	18:3	×	×	×
MGTS 18:4	494.3473	18:4	×	×	×
MGTS 18:5	492.3317	18:5			×
MGTS 19:1	514.4115	*	×		
MGTS 20:0	530.4423	*			×
MGTS 20:1	528.4255	*			×
MGTS 20:3	524.3933	*			×
MGTS 20:4	522.3785	*	×		×
MGTS 20:5	520.3624	*	×	×	×
MGTS 22:0	558.4726	*			×
MGTS 22:1	556.4596	*			×
MGTS 22:3	552.4252	*			
MGTS 22:4	550.4095	**			
MGTS 22:5	548.3944	*			×
MGTS 22:6	546.3782	*			
MGTS 24:0	586.5038	*			×
**DGTA identified as [M+H]^+^**					
DGTA 28:0	656.5463	14:0/14:0		×	
DGTA 28:1	654.5305	**		×	
DGTA 30:0	684.5772	14:0_16:0		×	
DGTA 30:1	682.5616	**		×	
DGTA 30:3	678.5291	**		×	
DGTA 32:1	710.5924	14:0_18:1 and 16:0_16:1		×	
DGTA 32:2	708.5766	14:0_18:2		×	
DGTA 32:3	706.5596	14:0_18:3		×	
DGTA 32:4	704.5455	14:0_18:4 and 16:0_16:4		×	
DGTA 34:1	738.6232	16:0_18:1		×	
DGTA 34:2	736.6083	16:0_18:2		×	
DGTA 34:3	734.5915	16:0_18:3		×	
DGTA 34:4	732.5768	16:0_18:4		×	
DGTA 34:5	730.5620	16:1_18:4			
DGTA 34:6	728.5433	*		×	
DGTA 34:7	726.5305	**		×	
DGTA 36:1	766.6528	**		×	
DGTA 36:2	764.6399	18:1/18:1		×	
DGTA 36:3	762.6219	**		×	
DGTA 36:4	760.6069	**		×	
DGTA 36:5	758.5923	16:0_20:5		×	
DGTA 36:6	756.5761	**		×	
DGTA 36:7	754.5594	*		×	
DGTA 36:8	752.5432	**		×	
DGTA 38:1	794.6881	**		×	
DGTA 38:5	786.6229	18:1_20:4		×	
DGTA 38:6	784.6072	18:2_20:4		×	
DGTA 38:7	782.5913	*		×	
DGTA 38:8	780.5796	**		×	
DGTA 40:6	812.6406	18:2_22:4		×	
DGTA 40:7	810.6237	**		×	
DGTA 40:8	808.6075	*		×	
DGTA 40:9	806.5912	*		×	
DGTA 40:10	804.5748	**		×	
DGTA 42:5	842.6871	**		×	
DGTA 42:11	830.5896	**		×	
**MGTA identified as [M+H]^+^**					
MGTA 14:0	446.3472	14:0		×	
MGTA 16:0	474.3786	*		×	
MGTA 16:1	472.3632	*		×	
MGTA 18:1	500.3945	*		×	
MGTA 18:2	498.3788	*		×	
MGTA 18:3	496.3620	18:3		×	
MGTA 18:4	494.3478	**		×	
MGTA 20:1	528.4265	*		×	
MGTA 20:2	526.4101	**		×	
MGTA 20:3	524.3950	**		×	
MGTA 20:4	522.3779	*		×	
MGTA 20:5	520.3630	**		×	
MGTA 22:1	556.4580	*		×	

**Table 7:** Lipid extract concentration (μg mL^−1^) of the algae blend (BLEND) that provided 50% (IC_50_) inhibition of the ABTS^•+^, and 35% (IC_35_) and 20% (IC_20_) inhibition of the DPPH^•^, in comparison with the values reported in the literature for the individual algae species—*Chlorella vulgaris*, *Fucus vesiculosus*, and *Ulva rigida*. Values are expressed as the mean ± SD (*n* = 3).

Assay		BLEND	*Chlorella vulgaris*		*Fucus vesiculosus*		*Ulva rigida*	
ABTS^•+^	IC_50_	140.01 ± 4.40	51	[7,30]	27	[17]	31	[17]
	TE ^1^	126.56 ± 3.91	368		507		501	
DPPH^•^	IC_35_	230.62 ± 3.30	–		–		–	
	IC_20_	132.67 ± 9.91	51	[7]	106	[17]	121	[17]
	TE ^1^	74.09 ± 1.07	192		90		88	

^1^ TE: trolox equivalents [μmol of Trolox g^−1^].

## Data Availability

Not applicable.

## Supplementary Materials

The following supporting information can be downloaded at: https://www.mdpi.com/article/10.3390/life13010231/s1, Figure S1. Calibration curve for the ABTS^•+^ scavenging assay as generated by measuring the absorbance of the reaction medium at 734 nm after 120 min. Trolox was used as standard. Figure S2. Calibration curve for the DPPH^•^ scavenging assay as generated by measuring the absorbance of the reaction medium at 517 nm after 120 min. Trolox was used as standard. Table S1: Glycolipids identified in the polar lipidome of the algae blend (BLEND) by high resolution HILIC-ESI-MS and MS/MS. C carbons, N number of double bonds. * Lipid species identified based on the polar head fragment, calculated mass and retention time. ** Lipid species identified based on calculated mass and retention time. Table S2: Phospholipids identified in the polar lipidome of the algae blend (BLEND) by high resolution HILIC–ESI–MS and MS/MS. C carbons, N number of double bonds. * Lipid species identified based on the polar head fragment, calculated mass and retention time. ** Lipid species identified based on calculated mass and retention time. Table S3: Betaine lipids identified in the polar lipidome of the algae blend (BLEND) by high resolution HILIC-ESI-MS and MS/MS. C carbons, N number of double bonds. * Lipid species identified based on the polar head fragment, calculated mass and retention time. ** Lipid species identified based on calculated mass and retention time.
