# Supplementary material for: Lipidomic Characterization and Antioxidant Activity of Macro- and Microalgae Blend
Source: Life (Basel). 2023 Jan 13;13(1):231. doi: 10.3390/life13010231 (PMC9865938; doi:10.3390/life13010231)
Supplement: Supplementary file 1 [file life-13-00231-s001.zip › life-2133084-supplementary.pdf]

Supplementary Information

Lipidomic Characterization and Antioxidant Activity of Macro- And Microalgae Blend

Francisca Marques [1,2], Diana Lopes [1,2], Tiago Conde [1,2], Tânia Melo [1,2], Joana Silva [3], Maria Helena Abreu [4], Pedro Domingues [2] and Maria Rosário Domingues [1,2,*]

[1] CESAM—Centre for Environmental and Marine Studies, Department of Chemistry, University of Aveiro, Santiago University Campus, 3810-193 Aveiro, Portugal

[2] Mass Spectrometry Centre, LAQV-REQUIMTE, Department of Chemistry, University of Aveiro, Santiago University Campus, 3810-193 Aveiro, Portugal

[3] Allmicroalgae Natural Products S.A., R&D Department, Rua 25 de Abril 19, 2445-287 Pataias, Portugal

[4] ALGAplus—Production and Trading of Seaweed and Derived Products Ltd., 3830-196 Ílhavo, Portugal

* Correspondence: mrd@ua.pt

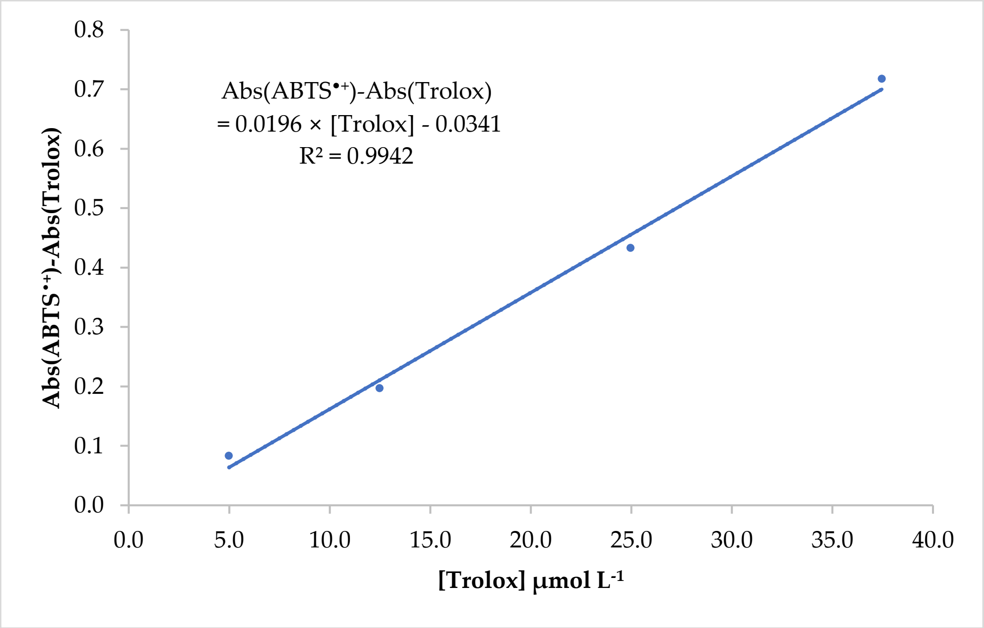

Figure S1. Calibration curve for the $ABTS^{•+}$ scavenging assay as generated by measuring the absorbance of the reaction medium at 734 nm after 120 min. Trolox was used as standard.

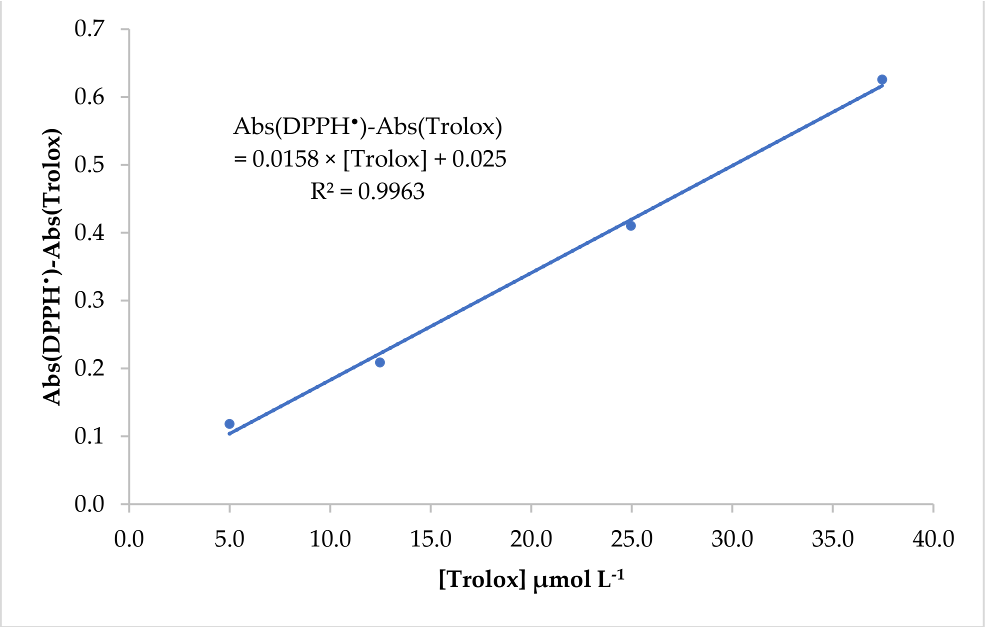

Figure S2. Calibration curve for the DPPH• scavenging assay as generated by measuring the absorbance of the reaction medium at 517 nm after 120 min. Trolox was used as standard.

Table S1. Glycolipids identified in the polar lipidome of the algae blend (BLEND) by high resolution HILIC–ESI–MS and MS/MS. C carbons, N number of double bonds. *Lipid species identified based on the polar head fragment, calculated mass and retention time. **Lipid species identified based on calculated mass and retention time.

Lipid species (C:N)	Calculated *m/z*	Observed *m/z*	Error (ppm)	Fatty acyl chains (C:N)	Formula
		**MGDG identified as $[M+NH_4]^+$**			
MGDG 30:4	712.5000	712.4980	-2.77	**	$C_{39}H_{70}O_{10}N$
MGDG 32:1	746.5782	746.5765	-2.30	**	$C_{41}H_{80}O_{10}N$
MGDG 32:2	744.5626	744.5604	-2.92	**	$C_{41}H_{78}O_{10}N$
MGDG 32:3	742.5469	742.5447	-3.00	**	$C_{41}H_{76}O_{10}N$
MGDG 32:4	740.5313	740.5289	-3.20	**	$C_{41}H_{74}O_{10}N$
MGDG 32:5	738.5156	738.5144	-1.66	16:2_16:3	$C_{41}H_{72}O_{10}N$
MGDG 32:6	736.5000	736.4986	-1.87	16:2_16:4 and 16:3/16:3	$C_{41}H_{70}O_{10}N$
MGDG 34:1	774.6095	774.6058	-4.81	**	$C_{43}H_{84}O_{10}N$
MGDG 34:2	772.5939	772.5925	-1.78	**	$C_{43}H_{82}O_{10}N$
MGDG 34:3	770.5782	770.5747	-4.57	16:0_18:3 and 16:3_18:0	$C_{43}H_{80}O_{10}N$
MGDG 34:4	768.5626	768.5607	-2.44	16:1_18:3 and 16:2_18:2 and 16:3_18:1	$C_{43}H_{78}O_{10}N$
MGDG 34:5	766.5469	766.5441	-3.68	**	$C_{43}H_{76}O_{10}N$
MGDG 34:6	764.5313	764.5302	-1.40	16:3_18:3	$C_{43}H_{74}O_{10}N$
MGDG 34:8	760.5000	760.4984	-2.07	16:4_18:4	$C_{43}H_{70}O_{10}N$
MGDG 34:9	758.4843	758.4871	3.66	*	$C_{43}H_{68}O_{10}N$
MGDG 35:1	784.5939	784.5930	-1.11	**	$C_{44}H_{82}O_{10}N$
MGDG 35:6	778.5464	778.5442	-2.81	17:3_18:3	$C_{44}H_{72}O_{10}N$
MGDG 36:4	796.5939	796.5921	-2.23	18:1_18:3 and 18:2/18:2	$C_{45}H_{82}O_{10}N$
MGDG 36:5	794.5782	794.5766	-2.04	**	$C_{45}H_{80}O_{10}N$
MGDG 36:6	792.5626	792.5615	-1.36	*	$C_{45}H_{78}O_{10}N$
MGDG 36:7	790.5469	790.5487	2.25	**	$C_{45}H_{76}O_{10}N$

MGDG 36:8	788.5313	788.5333	2.57	**	$C_{45}H_{74}O_{10}N$
MGDG 36:9	786.5156	786.5195	4.93	**	$C_{45}H_{72}O_{10}N$
MGDG 38:5	822.6095	822.6093	-0.27	**	$C_{47}H_{84}O_{10}N$
MGDG 38:6	820.5939	820.5954	1.86	**	$C_{47}H_{82}O_{10}N$
MGDG 38:7	818.5782	818.5770	-1.50	**	$C_{47}H_{80}O_{10}N$
MGDG 38:8	816.5626	816.5594	-3.89	**	$C_{47}H_{78}O_{10}N$
MGDG 38:9	814.5469	814.5447	-2.73	**	$C_{47}H_{76}O_{10}N$
MGDG 40:9	842.5782	842.5744	-4.54	**	$C_{49}H_{80}O_{10}N$
**MGMG identified as $[M+NH_4]^+$**					
MGMG 16:0	510.3642	510.3633	-1.78	**	$C_{25}H_{52}O_9N$
MGMG 16:1	508.3486	508.3484	-0.31	**	$C_{25}H_{50}O_9N$
MGMG 16:2	506.3329	506.3328	-0.22	**	$C_{25}H_{48}O_9N$
MGMG 16:3	504.3173	504.3172	-0.12	**	$C_{25}H_{46}O_9N$
MGMG 16:4	502.3016	502.3006	-2.01	**	$C_{25}H_{44}O_9N$
MGMG 18:1	536.3799	536.3795	-0.67	**	$C_{27}H_{54}O_9N$
MGMG 18:2	534.3642	534.3642	-0.02	**	$C_{27}H_{52}O_9N$
MGMG 18:3	532.3486	532.3479	-1.24	**	$C_{27}H_{50}O_9N$
MGMG 18:4	530.3329	530.3320	-1.71	**	$C_{27}H_{48}O_9N$
MGMG 20:4	558.3642	558.3630	-2.17	**	$C_{29}H_{52}O_9N$
MGMG 20:5	556.3486	556.3481	-0.83	**	$C_{29}H_{50}O_9N$
**DGDG identified as $[M+NH_4]^+$**					
DGDG 28:0	854.5841	854.5833	-0.93	*	$C_{43}H_{84}O_{15}N$
DGDG 30:0	882.6154	882.6153	-0.11	*	$C_{45}H_{88}O_{15}N$
DGDG 32:0	910.6467	910.6476	0.99	**	$C_{47}H_{92}O_{15}N$
DGDG 32:1	908.6310	908.6297	-1.48	14:0_18:1	$C_{47}H_{90}O_{15}N$
DGDG 32:2	906.6154	906.6156	0.22	14:0_18:2	$C_{47}H_{88}O_{15}N$
DGDG 32:3	904.5997	904.6006	0.94	14:0_18:3 and 16:0_16:3	$C_{47}H_{86}O_{15}N$
DGDG 32:4	902.5841	902.5836	-0.55	14:0_18:4 and 16:1_16:3 and 16:2/16:2	$C_{47}H_{84}O_{15}N$
DGDG 32:5	900.5684	900.5676	-0.94	16:2_16:3 and 16:1_16:4	$C_{47}H_{82}O_{15}N$
DGDG 34:1	936.6623	936.6602	-2.29	16:0_18:1 and 16:1_18:0	$C_{49}H_{94}O_{15}N$
DGDG 34:2	934.6467	934.6447	-2.14	16:0_18:2 and 16:1_18:1 and 17:0_17:2	$C_{49}H_{92}O_{15}N$
DGDG 34:3	932.6310	932.6294	-1.77	16:0_18:3 and 16:1_18:2 and 16:2_18:1	$C_{49}H_{90}O_{15}N$
DGDG 34:4	930.6154	930.6140	-1.50	16:0_18:4 and 16:1_18:3 and 16:2_18:2 and 16:3_18:1	$C_{49}H_{88}O_{15}N$
DGDG 34:5	928.5997	928.5969	-3.07	16:2_18:3 and 16:3_18:2	$C_{49}H_{86}O_{15}N$
DGDG 34:6	926.5841	926.5831	-1.08	16:3_18:3	$C_{49}H_{84}O_{15}N$
DGDG 34:7	924.5684	924.5653	-3.41	**	$C_{49}H_{82}O_{15}N$
DGDG 34:8	922.5528	922.5515	-1.41	16:4_18:4	$C_{49}H_{80}O_{15}N$
DGDG 35:1	950.6780	950.6733	-4.94	**	$C_{50}H_{96}O_{15}N$
DGDG 35:2	948.6623	948.6634	1.11	**	$C_{50}H_{94}O_{15}N$
DGDG 35:3	946.6467	946.6464	-0.32	17:0_18:3	$C_{50}H_{92}O_{15}N$
DGDG 36:1	964.6936	964.6890	-4.82	*	$C_{51}H_{98}O_{15}N$
DGDG 36:2	962.6780	962.6748	-3.32	**	$C_{51}H_{96}O_{15}N$
DGDG 36:3	960.6623	960.6590	-3.49	**	$C_{51}H_{94}O_{15}N$
DGDG 36:4	958.6467	958.6438	-3.02	**	$C_{51}H_{92}O_{15}N$
DGDG 36:5	956.6310	956.6301	-0.99	**	$C_{51}H_{90}O_{15}N$
DGDG 36:6	954.6154	954.6157	0.32	18:3/18:3	$C_{51}H_{88}O_{15}N$
DGDG 36:7	952.5997	952.6031	3.52	**	$C_{51}H_{86}O_{15}N$
DGDG 36:8	950.5841	950.5872	3.26	**	$C_{51}H_{84}O_{15}N$
DGDG 36:9	948.5684	948.5730	4.80	**	$C_{51}H_{82}O_{15}N$
DGDG 38:5	984.6623	984.6586	-3.81	**	$C_{53}H_{94}O_{15}N$

DGDG 38:6	982.6467	982.6450	-1.73	18:1_20:5	$C_{53}H_{92}O_{15}N$
DGDG 38:7	980.6310	980.6286	-2.50	**	$C_{53}H_{90}O_{15}N$
DGDG 38:8	978.6154	978.6140	-1.43	18:3_20:5	$C_{53}H_{88}O_{15}N$
DGDG 38:9	976.5997	976.6000	0.26	**	$C_{53}H_{86}O_{15}N$
**DGMG identified as $[M+NH_4]^+$**					
DGMG 16:0	672.4170	672.4163	-1.09	**	$C_{31}H_{62}O_{14}N$
DGMG 16:1	670.4014	670.4004	-1.47	**	$C_{31}H_{60}O_{14}N$
DGMG 16:4	664.3544	664.3538	-0.95	**	$C_{31}H_{54}O_{14}N$
DGMG 18:1	698.4327	698.4322	-0.69	**	$C_{33}H_{64}O_{14}N$
DGMG 18:2	696.4170	696.4172	0.24	*	$C_{33}H_{62}O_{14}N$
DGMG 18:3	694.4014	694.4015	0.17	**	$C_{33}H_{60}O_{14}N$
DGMG 18:4	692.3857	692.3839	-2.65	**	$C_{33}H_{58}O_{14}N$
DGMG 20:5	718.4014	718.4000	-1.93	**	$C_{35}H_{60}O_{14}N$
**SQDG identified as $[M–H]^-$**					
SQDG 28:0	737.4510	737.4519	1.25	14:0/14:0	$C_{37}H_{69}O_{12}S$
SQDG 30:0	765.4823	765.4831	1.08	14:0_16:0	$C_{39}H_{73}O_{12}S$
SQDG 30:1	763.4666	763.4671	0.62	*	$C_{39}H_{71}O_{12}S$
SQDG 30:4	757.4197	757.4215	2.41	14:0_16:4	$C_{39}H_{65}O_{12}S$
SQDG 31:1	777.4828	777.4821	-0.89	15:0_16:1	$C_{40}H_{74}O_{12}S$
SQDG 32:0	793.5136	793.5142	0.79	16:0/16:0	$C_{41}H_{77}O_{12}S$
SQDG 32:1	791.4979	791.4987	0.98	14:0_18:1	$C_{41}H_{75}O_{12}S$
SQDG 32:2	789.4823	789.4831	1.04	14:0_18:2 and 16:0_16:2	$C_{41}H_{73}O_{12}S$
SQDG 32:3	787.4666	787.4676	1.24	14:0_18:3 and 16:0_16:3 and 16:1_16:2	$C_{41}H_{71}O_{12}S$
SQDG 32:4	785.4510	785.4528	2.32	**	$C_{41}H_{69}O_{12}S$
SQDG 33:0	807.5292	807.5259	-4.12	**	$C_{42}H_{79}O_{12}S$
SQDG 33:1	805.5136	805.5113	-2.83	14:0_19:1 and 15:0_18:1 and 16:1_17:0	$C_{42}H_{77}O_{12}S$
SQDG 33:3	801.4828	801.4830	0.26	15:0_18:3	$C_{42}H_{73}O_{12}S$
SQDG 34:0	821.5449	821.5433	-1.92	14:0_20:0 and 16:0_18:0	$C_{43}H_{81}O_{12}S$
SQDG 34:1	819.5292	819.5302	1.19	16:0_18:1	$C_{43}H_{79}O_{12}S$
SQDG 34:2	817.5136	817.5118	-2.17	*	$C_{43}H_{77}O_{12}S$
SQDG 34:3	815.4979	815.4987	0.95	16:0_18:3	$C_{43}H_{75}O_{12}S$
SQDG 34:4	813.4823	813.4834	1.38	16:0_18:4	$C_{43}H_{73}O_{12}S$
SQDG 34:5	811.4666	811.4678	1.45	16:2_18:3	$C_{43}H_{71}O_{12}S$
SQDG 34:6	809.4510	809.4519	1.14	20:5_24:1	$C_{43}H_{69}O_{12}S$
SQDG 36:0	849.5762	849.5737	-2.92	*	$C_{45}H_{85}O_{12}S$
SQDG 36:1	847.5605	847.5602	-0.39	*	$C_{45}H_{83}O_{12}S$
SQDG 36:2	845.5449	845.5452	0.38	**	$C_{45}H_{81}O_{12}S$
SQDG 36:3	843.5292	843.5280	-1.45	**	$C_{45}H_{79}O_{12}S$
SQDG 36:4	841.5136	841.5137	0.15	18:2_18:2	$C_{45}H_{77}O_{12}S$
SQDG 36:5	839.4979	839.4970	-1.10	*	$C_{45}H_{75}O_{12}S$
SQDG 36:6	837.4823	837.4830	0.86	18:3_18:3	$C_{45}H_{73}O_{12}S$
SQDG 38:0	877.6075	877.6068	-0.77	**	$C_{47}H_{89}O_{12}S$
SQDG 38:1	875.5918	875.5922	0.43	14:0_24:1 and 16:0_22:1	$C_{47}H_{87}O_{12}S$
SQDG 38:5	867.5292	867.5301	1.01	**	$C_{47}H_{79}O_{12}S$
SQDG 40:1	903.6231	903.6240	0.97	16:0_24:1	$C_{49}H_{91}O_{12}S$
SQDG 42:1	931.6544	931.6566	2.33	**	$C_{51}H_{95}O_{12}S$
SQDG 42:8	917.5449	917.5426	-2.48	**	$C_{51}H_{81}O_{12}S$
SQDG 44:5	951.6231	951.6221	-1.08	**	$C_{53}H_{91}O_{12}S$
**SQMG identified as $[M–H]^-$**					
SQMG 16:0	555.2839	555.2843	0.70	**	$C_{25}H_{47}O_{11}S$
SQMG 16:4	547.2213	547.2230	3.08	**	$C_{25}H_{39}O_{11}S$

SQMG 18:1	581.2996	581.2994	-0.28	18:1	$C_{27}H_{49}O_{11}S$

Table S2. Phospholipids identified in the polar lipidome of the algae blend (BLEND) by high resolution HILIC–ESI–MS and MS/MS. C carbons, N number of double bonds. *Lipid species identified based on the polar head fragment, calculated mass and retention time. **Lipid species identified based on calculated mass and retention time.

Lipid species (C:N)	Calculated *m*/*z*	Observed *m*/*z*	Error (ppm)	Fatty acyl chains (C:N)	Formula
			**PC identified as [M+H]⁺**		
PC 30:0	706.5387	706.5389	0.31	**	$C_{38}H_{77}NO_8P$
PC 30:3	700.4917	700.4890	-3.90	**	$C_{38}H_{71}NO_8P$
PC 31:1	718.5381	718.5362	-2.63	15:0_16:1 and 15:1_16:0	$C_{39}H_{77}NO_8P$
PC 31:3	714.5068	714.5067	-0.10	15:0_16:3 and 15:1_16:2	$C_{39}H_{73}NO_8P$
PC 31:4	712.4912	712.4907	-0.72	**	$C_{39}H_{71}NO_8P$
PC 32:0	734.5700	734.5679	-2.83	**	$C_{40}H_{81}NO_8P$
PC 32:1	732.5543	732.5521	-3.05	16:0_16:1	$C_{40}H_{79}NO_8P$
PC 32:2	730.5387	730.5370	-2.30	16:0–16:2 and 16:1–16:1 and 14:0–18:2	$C_{40}H_{77}NO_8P$
PC 32:3	728.5230	728.5224	-0.87	16:0_16:3 and 16:1_16:2	$C_{40}H_{75}NO_8P$
PC 32:4	726.5074	726.5065	-1.21	16:1_16: and 16:2/16:2	$C_{40}H_{73}NO_8P$
PC 32:5	724.4917	724.4914	-0.46	16:2_16:3	$C_{40}H_{71}NO_8P$
PC 32:6	722.4755	722.4753	-0.30	16:3/16:3	$C_{40}H_{69}NO_8P$
PC 33:2	744.5538	744.5528	-1.30	**	$C_{41}H_{79}NO_8P$
PC 33:3	742.5381	742.5381	0.01	15:0_18:3 and 16:1_17:2 and 16:2_17:1 and 16:3_17:0	$C_{41}H_{77}NO_8P$
PC 33:4	740.5225	740.5223	-0.30	16:2_17:2 and 16:3_17:1 and 16:4_17:0	$C_{41}H_{75}NO_8P$
PC 33:5	738.5068	738.5068	0.04	**	$C_{41}H_{73}NO_8P$
PC 33:6	736.4917	736.4907	-1.40	16:3_17:3	$C_{41}H_{71}NO_8P$
PC 34:1	760.5856	760.5820	-4.78	16:0_18:1 and 16:1_18:0	$C_{42}H_{83}NO_8P$
PC 34:2	758.5700	758.5688	-1.56	*	$C_{42}H_{81}NO_8P$
PC 34:3	756.5543	756.5535	-1.10	16:0_18:3 and 16:1_18:2 and 16:2_18:1 and 16:3_18:0	$C_{42}H_{79}NO_8P$
PC 34:4	754.5387	754.5374	-1.70	16:0_18:4 and 16:1_18:3 and 16:2_18:2 and 16:3_18:1 and 16:4_18:0	$C_{42}H_{77}NO_8P$
PC 34:5	752.5230	752.5216	-1.90	16:1_18:4 and 16:2_18:3 and 16:3_18:2	$C_{42}H_{75}NO_8P$
PC 34:6	750.5074	750.5063	-1.44	16:3_18:3	$C_{42}H_{73}NO_8P$
PC 34:7	748.4917	748.4904	-1.78	**	$C_{42}H_{71}NO_8P$
PC 34:8	746.4761	746.4734	-3.59	**	$C_{42}H_{69}NO_8P$
PC 34:9	744.4604	744.4590	-1.92	*	$C_{42}H_{67}NO_8P$
PC 35:1	774.6013	774.5998	-1.91	17:0_18:1	$C_{43}H_{85}NO_8P$
PC 35:2	772.5851	772.5842	-1.14	17:0_18:2	$C_{43}H_{83}NO_8P$
PC 35:3	770.5694	770.5689	-0.65	17:0_18:3 and 17:1_18:2 and 17:2_18:3	$C_{43}H_{81}NO_8P$
PC 35:4	768.5538	768.5526	-1.52	17:1_18:3 and 17:2_18:2	$C_{43}H_{79}NO_8P$
PC 35:5	766.5381	766.5367	-1.81	17:2_18:3 and 17:3_18:2	$C_{43}H_{77}NO_8P$
PC 35:6	764.5225	764.5212	-1.73	**	$C_{43}H_{75}NO_8P$
PC 35:7	762.5068	762.5049	-2.45	*	$C_{43}H_{73}NO_8P$
PC 35:9	758.4755	758.4750	-0.69	*	$C_{43}H_{69}NO_8P$
PC 36:2	786.6013	786.6020	0.91	16:1_20:1 and 17:1_19:1 and 18:0_18:2 and 18:1/18:1	$C_{44}H_{85}NO_8P$
PC 36:3	784.5856	784.5842	-1.83	18:0_18:3 and 18:1_18:2	$C_{44}H_{83}NO_8P$
PC 36:4	782.5700	782.5690	-1.25	16:1_20:3 and 16:2_20:2 and 18:1_18:3 and 18:2/18:2	$C_{44}H_{81}NO_8P$

PC 36:5	780.5538	780.5535	-0.35	16:0_20:5 and 16:1_20:4 and 18:2_18:3	$C_{44}H_{79}NO_8P$
PC 36:6	778.5387	778.5372	-1.90	16:3_20:3 and 18:2_18:4 and 18:3/18:3	$C_{44}H_{77}NO_8P$
PC 36:7	776.5230	776.5212	-2.36	**	$C_{44}H_{75}NO_8P$
PC 36:8	774.5074	774.5038	-4.62	**	$C_{44}H_{73}NO_8P$
PC 36:9	772.4917	772.4887	-3.92	**	$C_{44}H_{71}NO_8P$
PC 37:3	798.6007	798.6003	-0.51	**	$C_{45}H_{85}NO_8P$
PC 37:4	796.5851	796.5851	0.03	18:3_19:1	$C_{45}H_{83}NO_8P$
PC 38:2	814.6326	814.6302	-2.92	18:1_20:1 and 18:2_20:0	$C_{46}H_{89}NO_8P$
PC 38:3	812.6169	812.6163	-0.78	18:1_20:2 and 18:2_20:1 and 18:3_20:0	$C_{46}H_{87}NO_8P$
PC 38:4	810.6013	810.6008	-0.59	*	$C_{46}H_{85}NO_8P$
PC 38:5	808.5856	808.5827	-3.63	18:2_20:3 and 18:3_20:2	$C_{46}H_{83}NO_8P$
PC 38:6	806.5694	806.5663	-3.84	18:2_20:4 and 18:3_20:3	$C_{46}H_{81}NO_8P$
PC 38:7	804.5543	804.5525	-2.28	**	$C_{46}H_{79}NO_8P$
PC 38:8	802.5387	802.5367	-2.47	**	$C_{46}H_{75}NO_8P$
PC 38:9	800.5230	800.5199	-3.91	**	$C_{46}H_{73}NO_8P$
PC 39:9	814.5381	814.5347	-4.16	**	$C_{47}H_{77}NO_8P$
PC 40:6	834.6013	834.5972	-4.89	**	$C_{48}H_{85}NO_8P$
PC 40:7	832.5856	832.5833	-2.80	**	$C_{48}H_{83}NO_8P$
PC 40:8	830.5700	830.5668	-3.83	**	$C_{48}H_{81}NO_8P$
PC 40:9	828.5543	828.5521	-2.69	**	$C_{48}H_{79}NO_8P$
PC 40:10	826.5387	826.5352	-4.21	**	$C_{48}H_{77}NO_8P$
PC 44:2	898.7265	898.7245	-2.21	**	$C_{52}H_{101}NO_8P$
PC 44:3	896.7108	896.7108	-0.04	**	$C_{52}H_{99}NO_8P$
**LPC identified as [M+H]+**					
LPC 14:0	468.3090	468.3085	-1.10	**	$C_{22}H_{47}NO_7P$
LPC 16:0	496.3403	496.3395	-1.64	16:0	$C_{24}H_{51}NO_7P$
LPC 16:1	494.3247	494.3241	-1.15	16:1	$C_{24}H_{49}NO_7P$
LPC 16:2	492.3090	492.3083	-1.46	16:2	$C_{24}H_{47}NO_7P$
LPC 16:3	490.2934	490.2925	-1.77	16:3	$C_{24}H_{45}NO_7P$
LPC 17:0	510.3554	510.3551	-0.61	17:0	$C_{25}H_{53}NO_7P$
LPC 17:1	508.3398	508.3391	-1.40	17:1	$C_{25}H_{51}NO_7P$
LPC 17:3	504.3085	504.3075	-1.98	17:3	$C_{25}H_{47}NO_7P$
LPC 18:0	524.3716	524.3709	-1.37	18:0	$C_{26}H_{55}NO_7P$
LPC 18:1	522.3560	522.3563	0.64	**	$C_{26}H_{53}NO_7P$
LPC 18:2	520.3403	520.3400	-0.61	18:2	$C_{26}H_{51}NO_7P$
LPC 18:3	518.3247	518.3237	-1.87	18:3	$C_{26}H_{49}NO_7P$
LPC 19:1	536.3711	536.3723	2.26	19:1	$C_{27}H_{55}NO_7P$
LPC 19:2	534.3554	534.3546	-1.52	19:2	$C_{27}H_{53}NO_7P$
LPC 20:1	550.3873	550.3869	-0.67	20:1	$C_{28}H_{57}NO_7P$
LPC 20:2	548.3716	548.3696	-3.68	20:2	$C_{28}H_{55}NO_7P$
LPC 20:3	546.3560	546.3536	-4.33	**	$C_{28}H_{53}NO_7P$
LPC 20:4	544.3398	544.3387	-1.98	**	$C_{28}H_{51}NO_7P$
LPC 20:5	542.3247	542.3221	-4.73	20:5	$C_{28}H_{49}NO_7P$
LPC 22:5	570.3560	570.3542	-3.10	**	$C_{30}H_{53}NO_7P$
LPC 22:6	568.3403	568.3385	-3.20	**	$C_{30}H_{51}NO_7P$
**PE identified as [M–H]–**					
PE 28:1	632.4291	632.4302	1.69	14:0_14:1	$C_{33}H_{63}NO_8P$
PE 29:0	648.4604	648.4611	1.03	14:0_15:0	$C_{34}H_{67}NO_8P$
PE 29:1	646.4448	646.4456	1.27	14:1_15:0	$C_{34}H_{65}NO_8P$
PE 30:0	662.4761	662.4770	1.39	15:0/15:0	$C_{35}H_{69}NO_8P$
PE 30:1	660.4604	660.4612	1.16	14:0_16:1 and 14:1_16:0	$C_{35}H_{67}NO_8P$

PE 30:2	658.4448	658.4467	2.91	14:0_16:2	$C_{35}H_{65}NO_8P$
PE 30:3	656.4291	656.4320	4.37	**	$C_{35}H_{63}NO_8P$
PE 31:1	674.4761	674.4768	1.06	15:0_16:1	$C_{36}H_{69}NO_8P$
PE 31:2	672.4604	672.4615	1.59	15:1_16:1	$C_{36}H_{67}NO_8P$
PE 31:3	670.4448	670.4462	2.12	15:0_16:3	$C_{36}H_{65}NO_8P$
PE 32:1	688.4917	688.4923	0.82	16:0_16:1	$C_{37}H_{71}NO_8P$
PE 32:2	686.4761	686.4768	1.05	16:0_16:2 and 16:1/16:1	$C_{37}H_{69}NO_8P$
PE 32:3	684.4604	684.4612	1.12	14:0_18:3 and 16:0_16:3 and 16:1_16:2	$C_{37}H_{67}NO_8P$
PE 32:4	682.4448	682.4463	2.22	14:1_18:3 and 16:1_16:3 and 16:2/16:2	$C_{37}H_{65}NO_8P$
PE 32:5	680.4291	680.4296	0.69	**	$C_{37}H_{63}NO_8P$
PE 32:6	678.4135	678.4140	0.76	*	$C_{37}H_{61}NO_8P$
PE 33:1	702.5074	702.5079	0.74	**	$C_{38}H_{73}NO_8P$
PE 33:2	700.4917	700.4923	0.81	15:0_18:2 and 16:1_17:1 and 16:2_17:0	$C_{38}H_{71}NO_8P$
PE 33:3	698.4761	698.4770	1.31	15:0_18:3 and 16:3_17:0	$C_{38}H_{69}NO_8P$
PE 34:1	716.5230	716.5203	-3.81	16:0_18:1	$C_{39}H_{75}NO_8P$
PE 34:2	714.5074	714.5076	0.31	16:0_18:2 and 16:1_18:1	$C_{39}H_{73}NO_8P$
PE 34:3	712.4917	712.4924	0.94	16:0_18:3 and 16:1_18:2 and 16:2_18:1	$C_{39}H_{71}NO_8P$
PE 34:4	710.4761	710.4767	0.87	16:1_18:3 and 16:2_18:2 and 16:3_18:1	$C_{39}H_{69}NO_8P$
PE 34:5	708.4604	708.4613	1.23	16:2_18:3 and 16:3_18:2	$C_{39}H_{67}NO_8P$
PE 34:6	706.4448	706.4456	1.16	16:3_18:3	$C_{39}H_{65}NO_8P$
PE 35:2	728.5230	728.5236	0.78	17:0_18:2 and 17:1_18:1	$C_{40}H_{75}NO_8P$
PE 35:3	726.5074	726.5079	0.71	17:0_18:3 and 17:1_18:2 and 17:2_18:1	$C_{40}H_{73}NO_8P$
PE 35:4	724.4917	724.4924	0.92	17:0_18:4 and 17:1_18:3 and 17:2_18:2	$C_{40}H_{71}NO_8P$
PE 35:5	722.4761	722.4765	0.58	17:2_18:3	$C_{40}H_{69}NO_8P$
PE 35:6	720.4604	720.4610	0.79	17:3_18:3	$C_{40}H_{67}NO_8P$
PE 36:2	742.5387	742.5387	0.02	18:0_18:2 and 18:1/18:1	$C_{41}H_{77}NO_8P$
PE 36:3	740.5230	740.5227	-0.45	**	$C_{41}H_{75}NO_8P$
PE 36:4	738.5074	738.5090	2.19	18:1_18:3 and 18:2/18:2	$C_{41}H_{73}NO_8P$
PE 36:5	736.4917	736.4923	0.77	*	$C_{41}H_{71}NO_8P$
PE 36:6	734.4761	734.4769	1.11	18:3/18:3	$C_{41}H_{69}NO_8P$
PE 37:3	754.5387	754.5374	-1.70	18:2_19:1	$C_{42}H_{77}NO_8P$
PE 37:4	752.5230	752.5234	0.49	18:3_19:1	$C_{42}H_{75}NO_8P$
PE 37:5	750.5074	750.5075	0.16	18:3_19:2	$C_{42}H_{73}NO_8P$
PE 38:2	770.5700	770.5696	-0.50	18:1_20:1 and 18:2_20:0	$C_{43}H_{81}NO_8P$
PE 38:3	768.5543	768.5545	0.22	18:1_20:2 and 18:2_20:1 and 18:3_20:0	$C_{43}H_{79}NO_8P$
PE 38:4	766.5387	766.5389	0.28	18:2_20:2 and 18:3_20:1	$C_{43}H_{77}NO_8P$
PE 38:5	764.5230	764.5219	-1.48	*	$C_{43}H_{75}NO_8P$
PE 38:7	760.4917	760.4909	-1.09	*	$C_{43}H_{71}NO_8P$
PE 40:8	786.5074	786.5082	1.04	20:4/20:4	$C_{45}H_{73}NO_8P$
PE 40:9	784.4917	784.4915	-0.30	*	$C_{45}H_{71}NO_8P$
PE 40:11	780.4604	780.4598	-0.81	*	$C_{45}H_{67}NO_8P$
PE 42:2	826.6326	826.6337	1.35	**	$C_{47}H_{89}NO_8P$
**LPE identified as [M–H]⁻**					
LPE 14:0	424.2464	424.2469	1.14	*	$C_{19}H_{39}NO_7P$
LPE 15:0	438.2621	438.2626	1.22	*	$C_{20}H_{41}NO_7P$
LPE 16:0	452.2777	452.2783	1.29	*	$C_{21}H_{43}NO_7P$
LPE 16:1	450.2621	450.2626	1.18	16:1	$C_{21}H_{41}NO_7P$
LPE 16:2	448.2464	448.2470	1.30	**	$C_{21}H_{39}NO_7P$
LPE 16:3	446.2308	446.2314	1.42	**	$C_{21}H_{37}NO_7P$
LPE 17:0	466.2934	466.2939	1.14	*	$C_{22}H_{45}NO_7P$
LPE 17:1	464.2777	464.2780	0.61	17:1	$C_{22}H_{43}NO_7P$

LPE 18:1	478.2934	478.2940	1.32	18:1	$C_{23}H_{45}NO_7P$
LPE 18:2	476.2777	476.2782	1.01	18:2	$C_{23}H_{43}NO_7P$
LPE 18:3	474.2621	474.2624	0.72	18:3	$C_{23}H_{41}NO_7P$
LPE 19:1	492.3090	492.3097	1.39	19:1	$C_{24}H_{47}NO_7P$
LPE 20:0	508.3403	508.3410	1.35	**	$C_{25}H_{51}NO_7P$
LPE 20:1	506.3247	506.3255	1.65	**	$C_{25}H_{49}NO_7P$
LPE 20:4	500.2777	500.2784	1.37	20:4	$C_{25}H_{43}NO_7P$
LPE 20:5	498.2621	498.2626	1.07	**	$C_{25}H_{41}NO_7P$
LPE 22:1	534.3560	534.3576	3.06	*	$C_{27}H_{53}NO_7P$
LPE 22:5	526.2934	526.2938	0.82	*	$C_{27}H_{45}NO_7P$
**PG identified as $[M–H]^-$**					
PG 28:1	663.4237	663.4221	-2.43	14:0_14:1	$C_{34}H_{64}O_{10}P$
PG 30:0	693.4707	693.4711	0.63	14:0_16:0	$C_{36}H_{70}O_{10}P$
PG 30:1	691.4550	691.4542	-1.18	**	$C_{36}H_{68}O_{10}P$
PG 32:0	721.5025	721.5026	0.14	16:0/16:0	$C_{38}H_{74}O_{10}P$
PG 32:1	719.4863	719.4870	0.95	16:0_16:1	$C_{38}H_{72}O_{10}P$
PG 32:2	717.4707	717.4714	1.03	16:0_16:2 and 16:1/16:1	$C_{38}H_{70}O_{10}P$
PG 32:3	715.4550	715.4552	0.26	*	$C_{38}H_{68}O_{10}P$
PG 32:4	713.4394	713.4409	2.15	14:0_18:3 and 16:1_16:3 and 16:2/16:2	$C_{38}H_{66}O_{10}P$
PG 32:5	711.4237	711.4210	-3.81	**	$C_{38}H_{64}O_{10}P$
PG 33:0	735.5176	735.5186	1.34	**	C39H77O10P
PG 33:1	733.5025	733.5025	0.00	15:0_18:1 and 16:0_17:1 and 16:1_17:0	$C_{39}H_{74}O_{10}P$
PG 33:2	731.4869	731.4868	-0.11	15:0_18:2 and 16:0_17:2 and 16:1_17:1	$C_{39}H_{72}O_{10}P$
PG 33:3	729.4707	729.4714	1.01	15:0_18:3 and 16:3_17:0	$C_{39}H_{70}O_{10}P$
PG 34:0	749.5333	749.5309	-3.15	14:0_20:0 and 16:0_18:0	$C_{40}H_{78}O_{10}P$
PG 34:1	747.5176	747.5154	-2.96	16:0_18:1 and 16:1_18:0	$C_{40}H_{76}O_{10}P$
PG 34:2	745.5020	745.5020	0.05	16:0_18:2 and 16:1_18:1	$C_{40}H_{74}O_{10}P$
PG 34:3	743.4863	743.4869	0.79	16:0_18:3 and 16:1_18:2	$C_{40}H_{72}O_{10}P$
PG 34:4	741.4707	741.4714	0.99	14:0_20:4 and 16:0_18:4 and 16:1_18:3 and 16:2_18:2	$C_{40}H_{70}O_{10}P$
PG 34:5	739.4556	739.4559	0.37	14:0_20:5 and 16:1_18:4 and 16:2_18:3 and 16:3_18:2 and 16:4_18:1	$C_{40}H_{68}O_{10}P$
PG 35:2	759.5182	759.5170	-1.57	14:0_21:2 and 17:0_18:2	$C_{41}H_{76}O_{10}P$
PG 35:3	757.5025	757.5018	-0.92	14:0_21:3 and 17:0_18:3	$C_{41}H_{74}O_{10}P$
PG 35:4	755.4869	755.4859	-1.30	17:1_18:3	$C_{41}H_{72}O_{10}P$
PG 35:6	751.4556	751.4556	-0.04	17:2_18:4	$C_{41}H_{68}O_{10}P$
PG 36:1	775.5489	775.5458	-4.01	**	$C_{42}H_{80}O_{10}P$
PG 36:2	773.5333	773.5334	0.18	18:0_18:2 and 18:1/18:1	$C_{42}H_{78}O_{10}P$
PG 36:3	771.5176	771.5174	-0.28	**	$C_{42}H_{76}O_{10}P$
PG 36:4	769.5020	769.5020	0.05	16:0_20:4 and 16:1_20:3 and 18:1_18:3 and 18:2/18:2	$C_{42}H_{74}O_{10}P$
PG 36:5	767.4869	767.4860	-1.12	**	C42H72O10P
PG 36:9	759.4237	759.4227	-1.33	*	C42H64O10P
PG 38:5	795.5182	795.5207	3.19	18:1_20:4 and 18:2_20:3 and 18:3_20:2	C44H76O10P
PG 40:11	811.4550	811.4561	1.34	*	$C_{46}H_{68}O_{10}P$
PG 42:11	839.4863	839.4862	-0.13	*	$C_{48}H_{72}O_{10}P$
PG 44:5	879.6115	879.6088	-3.08	**	$C_{50}H_{88}O_{10}P$
PG 46:5	907.6428	907.6429	0.10	**	$C_{52}H_{92}O_{10}P$
**LPG identified as $[M–H]^-$**					
LPG 16:0	483.2723	483.2744	4.35	16:0	$C_{22}H_{44}O_9P$
LPG 16:1	481.2566	481.2571	0.94	*	$C_{22}H_{42}O_9P$

LPG 18:1	509.2879	509.2893	2.65	**	$C_{24}H_{46}O_{9}P$
LPG 18:2	507.2723	507.2732	1.78	**	$C_{24}H_{44}O_{9}P$
LPG 18:3	505.2566	505.2573	1.29	18:3	$C_{24}H_{42}O_{9}P$
**PI identified as [M–H]⁻**					
PI 30:0	781.4873	781.4844	-3.65	**	C39H74O13P
PI 30:2	777.4554	777.4545	-1.17	14:0_16:2 and 14:1_16:1	$C_{39}H_{70}O_{13}P$
PI 32:3	803.4711	803.4719	1.05	**	$C_{41}H_{72}O_{13}P$
PI 34:0	837.5499	837.5458	-4.84	**	C43H82O13P
PI 34:1	835.5337	835.5308	-3.42	16:0_18:1	$C_{43}H_{80}O_{13}P$
PI 34:2	833.5180	833.5185	0.59	16:0_18:2 and 16:1_18:1	$C_{43}H_{78}O_{13}P$
PI 34:3	831.5024	831.5037	1.61	16:0_18:3 and 16:1_18:2	$C_{43}H_{76}O_{13}P$
PI 34:4	829.4867	829.4841	-3.14	16:0_18:4 and 16:1_18:3 and 16:2_18:2	$C_{43}H_{74}O_{13}P$
PI 34:5	827.4711	827.4712	0.17	14:0_20:5 and 16:1_18:4 and 16:2_18:3 and 16:3_18:2 and 16:4_18:1	$C_{43}H_{72}O_{13}P$
PI 35:2	847.5337	847.5334	-0.30	15:0_20:2 and 17:0_18:2	C44H80O13P
PI 36:1	863.5655	863.5620	-4.06	**	C45H84O13P
PI 36:2	861.5493	861.5478	-1.75	**	$C_{45}H_{82}O_{13}P$
PI 36:3	859.5337	859.5336	-0.07	16:0_20:3 and 16:1_20:2 and 16:2_20:1 and 18:0_18:3 and 18:2_18:1	$C_{45}H_{80}O_{13}P$
PI 36:4	857.5180	857.5166	-1.64	16:0_20:4 and 18:0_18:4 and 18:1_18:3 and 18:2/18:2	$C_{45}H_{78}O_{13}P$
PI 36:5	855.5024	855.5034	1.22	16:0_20:5 and 18:1_18:4 and 18:2_18:3	$C_{45}H_{76}O_{13}P$
PI 36:6	853.4867	853.4895	3.27	**	$C_{45}H_{74}O_{13}P$
PI 38:4	885.5493	885.5527	3.83	18:0_20:4 and 18:1_20:3 and 18:2_20:2 and 18:3_20:1	$C_{47}H_{82}O_{13}P$
PI 38:5	883.5337	883.5373	4.12	**	$C_{47}H_{80}O_{13}P$
PI 38:8	877.4873	877.4843	-3.37	**	C47H74O13P
PI 40:7	907.5342	907.5305	-4.08	**	C49H80O13P
PI 40:10	901.4867	901.4855	-1.34	**	$C_{49}H_{74}O_{13}P$

Table S3. Betaine lipids identified in the polar lipidome of the algae blend (BLEND) by high resolution HILIC–ESI–MS and MS/MS. C carbons, N number of double bonds. *Lipid species identified based on the polar head fragment, calculated mass and retention time. **Lipid species identified based on calculated mass and retention time.

Lipid species (C:N)	Calculated m/z	Observed m/z	Error (ppm)	Fatty acyl chains (C:N)	Formula
**DGTS identified as [M+H]⁺**					
DGTS 28:0	656.5465	656.5463	-0.35	*	$C_{38}H_{74}O_{7}N$
DGTS 30:0	684.5778	684.5781	0.40	14:0_16:0	$C_{40}H_{78}O_{7}N$
DGTS 30:1	682.5622	682.5614	-1.14	14:0_16:1	$C_{40}H_{76}O_{7}N$
DGTS 30:3	678.5309	678.5291	-2.62	14:0_16:3	$C_{40}H_{72}O_{7}N$
DGTS 30:4	676.5152	676.5144	-1.23	**	$C_{40}H_{70}O_{7}N$
DGTS 32:0	712.6091	712.6080	-1.54	16:0/16:0	$C_{42}H_{82}O_{7}N$
DGTS 32:1	710.5935	710.5924	-1.52	14:0_18:1 and 16:0_16:1	$C_{42}H_{80}O_{7}N$
DGTS 32:2	708.5778	708.5768	-1.45	14:0_18:2 and 16:0_16:2 and 16:1/16:1	$C_{42}H_{78}O_{7}N$
DGTS 32:3	706.5622	706.5599	-3.23	*	$C_{42}H_{76}O_{7}N$
DGTS 32:4	704.5465	704.5455	-1.46	14:0_18:4 and 16:0_16:4	$C_{42}H_{74}O_{7}N$
DGTS 32:6	700.5152	700.5140	-1.75	**	$C_{42}H_{70}O_{7}N$
DGTS 33:1	724.6091	724.6093	0.24	16:0_17:1	$C_{43}H_{82}O_{7}N$
DGTS 33:2	722.5935	722.5925	-1.35	16:0_17:2	$C_{43}H_{80}O_{7}N$
DGTS 33:3	720.5778	720.5790	1.63	15:0_18:3	$C_{43}H_{78}O_{7}N$

DGTS 33:4	718.5622	718.5612	-1.36	15:0_18:4	$C_{43}H_{76}O_7N$
DGTS 34:1	738.6248	738.6232	-2.14	16:0_18:1	$C_{44}H_{84}O_7N$
DGTS 34:2	736.6091	736.6066	-3.43	16:0_18:2 and 16:1_18:1	$C_{44}H_{82}O_7N$
DGTS 34:3	734.5935	734.5915	-2.69	16:0_18:3	$C_{44}H_{80}O_7N$
DGTS 34:4	732.5778	732.5768	-1.40	16:0_18:4	$C_{44}H_{78}O_7N$
DGTS 34:5	730.5622	730.5608	-1.89	16:1_18:4 and 16:2_18:3	$C_{44}H_{76}O_7N$
DGTS 34:6	728.5465	728.5463	-0.31	*	$C_{44}H_{74}O_7N$
DGTS 34:7	726.5309	726.5286	-3.14	16:4_18:3	$C_{44}H_{72}O_7N$
DGTS 34:8	724.5152	724.5159	0.93	16:4_18:4	$C_{44}H_{70}O_7N$
DGTS 35:4	746.5935	746.5931	-0.51	15:0_20:4 and 17:1_18:3	$C_{45}H_{80}O_7N$
DGTS 35:5	744.5778	744.5763	-2.05	17:1_18:4	$C_{45}H_{78}O_7N$
DGTS 36:1	766.6561	766.6528	-4.28	16:0_20:1 and 18:0_18:1	$C_{46}H_{88}O_7N$
DGTS 36:2	764.6404	764.6399	-0.69	16:0_20:2 and 18:1/18:1	$C_{46}H_{86}O_7N$
DGTS 36:3	762.6248	762.6231	-2.20	16:0_20:3	$C_{46}H_{84}O_7N$
DGTS 36:4	760.6091	760.6069	-2.93	16:0_20:4 and 18:1_18:3	$C_{46}H_{82}O_7N$
DGTS 36:5	758.5935	758.5923	-1.55	16:0_20:5 and 18:1_18:4 and 18:2_18:3	$C_{46}H_{80}O_7N$
DGTS 36:6	756.5778	756.5743	-4.66	18:3/18:3	$C_{46}H_{78}O_7N$
DGTS 36:7	754.5622	754.5594	-3.68	18:3_18:4	$C_{46}H_{76}O_7N$
DGTS 36:8	752.5465	752.5452	-1.77	18:4/18:4	$C_{46}H_{74}O_7N$
DGTS 36:9	750.5309	750.5328	2.56	**	$C_{46}H_{72}O_7N$
DGTS 36:10	748.5152	748.5142	-1.37	*	$C_{46}H_{70}O_7N$
DGTS 38:0	796.7030	796.7023	-0.92	**	$C_{48}H_{94}O_7N$
DGTS 38:1	794.6874	794.6881	0.91	18:1_20:0	$C_{48}H_{92}O_7N$
DGTS 38:3	790.6561	790.6542	-2.38	*	$C_{48}H_{88}O_7N$
DGTS 38:5	786.6248	786.6238	-1.24	16:0_22:5	$C_{48}H_{84}O_7N$
DGTS 38:6	784.6091	784.6072	-2.46	16:0_22:5	$C_{48}H_{82}O_7N$
DGTS 38:7	782.5935	782.5901	-4.32	**	$C_{48}H_{80}O_7N$
DGTS 38:8	780.5778	780.5744	-4.39	18:4_20:4	$C_{48}H_{78}O_7N$
DGTS 38:9	778.5622	778.5592	-3.83	18:4_20:5	$C_{48}H_{76}O_7N$
DGTS 38:10	776.5465	776.5439	-3.39	*	$C_{48}H_{74}O_7N$
DGTS 40:3	818.6874	818.6850	-2.91	**	$C_{50}H_{92}O_7N$
DGTS 40:4	816.6717	816.6710	-0.89	16:0_24:4 and 18:4_22:0	$C_{50}H_{90}O_7N$
DGTS 40:5	814.6561	814.6525	-4.39	**	$C_{50}H_{88}O_7N$
DGTS 40:6	812.6404	812.6402	-0.28	18:1_22:5	$C_{50}H_{86}O_7N$
DGTS 40:7	810.6248	810.6229	-2.32	**	$C_{50}H_{84}O_7N$
DGTS 40:8	808.6091	808.6066	-3.13	18:3_22:5	$C_{50}H_{82}O_7N$
DGTS 40:9	806.5935	806.5920	-1.83	18:4_22:5	$C_{50}H_{80}O_7N$
DGTS 40:10	804.5778	804.5748	-3.76	20:5/20:5	$C_{50}H_{78}O_7N$
DGTS 42:5	842.6874	842.6879	0.62	**	$C_{52}H_{92}O_7N$
DGTS 42:6	840.6717	840.6688	-3.48	**	$C_{52}H_{90}O_7N$
DGTS 42:7	838.6561	838.6531	-3.55	20:3_22:4	$C_{52}H_{88}O_7N$
DGTS 42:9	834.6248	834.6230	-2.13	**	$C_{52}H_{84}O_7N$
DGTS 42:10	832.6091	832.6071	-2.44	**	$C_{52}H_{82}O_7N$
DGTS 42:11	830.5935	830.5905	-3.59	**	$C_{54}H_{98}O_7N$
DGTS 44:5	870.7187	870.7184	-0.32	22:0_22:5	$C_{54}H_{96}O_7N$
DGTS 44:10	860.6404	860.6385	-2.24	22:5_22:5	$C_{54}H_{86}O_7N$
DGTS 44:12	856.6091	856.6055	-4.24	**	$C_{54}H_{82}O_7N$
**MGTS identified as $[M+H]^+$**					
MGTS 14:0	446.3482	446.3472	-2.16	14:0	$C_{24}H_{48}O_6N$
MGTS 15:0	460.3638	460.3635	-0.68	*	$C_{25}H_{50}O_6N$
MGTS 16:0	474.3795	474.3788	-1.40	*	$C_{26}H_{52}O_6N$

MGTS 16:1	472.3638	472.3633	-1.09	*	$C_{26}H_{50}O_6N$
MGTS 16:2	470.3482	470.3478	-0.77	*	$C_{26}H_{48}O_6N$
MGTS 16:3	468.3325	468.3343	3.81	*	$C_{26}H_{46}O_6N$
MGTS 16:4	466.3169	466.3162	-1.42	*	$C_{26}H_{44}O_6N$
MGTS 17:1	486.3795	486.3787	-1.57	*	$C_{27}H_{52}O_6N$
MGTS 17:3	482.3482	482.3462	-4.07	*	$C_{27}H_{48}O_6N$
MGTS 18:0	502.4108	502.4091	-3.31	*	$C_{28}H_{56}O_6N$
MGTS 18:1	500.3951	500.3941	-2.03	*	$C_{28}H_{54}O_6N$
MGTS 18:2	498.3795	498.3800	1.08	*	$C_{28}H_{52}O_6N$
MGTS 18:3	496.3638	496.3613	-4.98	18:3	$C_{28}H_{50}O_6N$
MGTS 18:4	494.3482	494.3473	-1.75	18:4	$C_{28}H_{48}O_6N$
MGTS 18:5	492.3325	492.3317	-1.65	18:5	$C_{28}H_{46}O_6N$
MGTS 19:1	514.4108	514.4115	1.43	*	$C_{29}H_{56}O_6N$
MGTS 20:0	530.4421	530.4423	0.44	*	$C_{30}H_{60}O_6N$
MGTS 20:1	528.4264	528.4255	-1.73	*	$C_{30}H_{58}O_6N$
MGTS 20:3	524.3951	524.3933	-3.46	*	$C_{30}H_{54}O_6N$
MGTS 20:4	522.3795	522.3785	-1.85	*	$C_{30}H_{52}O_6N$
MGTS 20:5	520.3638	520.3624	-2.72	*	$C_{30}H_{50}O_6N$
MGTS 22:0	558.4734	558.4726	-1.37	*	$C_{32}H_{64}O_6N$
MGTS 22:1	556.4577	556.4596	3.39	*	$C_{32}H_{62}O_6N$
MGTS 22:3	552.4264	552.4252	-2.20	*	$C_{32}H_{58}O_6N$
MGTS 22:4	550.4108	550.4095	-2.30	**	$C_{32}H_{56}O_6N$
MGTS 22:5	548.3951	548.3944	-1.30	*	$C_{32}H_{54}O_6N$
MGTS 22:6	546.3795	546.3782	-2.31	*	$C_{32}H_{52}O_6N$
MGTS 24:0	586.5047	586.5038	-1.47	*	$C_{34}H_{68}O_6N$
**DGTA identified as $[M+H]^+$**					
DGTA 28:0	656.5465	656.5463	-0.35	14:0/14:0	$C_{38}H_{74}O_7N$
DGTA 28:1	654.5309	654.5305	-0.58	**	$C_{38}H_{72}O_7N$
DGTA 30:0	684.5778	684.5772	-0.92	14:0_16:0	$C_{40}H_{78}O_7N$
DGTA 30:1	682.5622	682.5616	-0.85	**	$C_{40}H_{76}O_7N$
DGTA 30:3	678.5309	678.5291	-2.62	**	$C_{40}H_{72}O_7N$
DGTA 32:1	710.5935	710.5924	-1.52	14:0_18:1 and 16:0_16:1	$C_{42}H_{80}O_7N$
DGTA 32:2	708.5778	708.5766	-1.73	14:0_18:2	$C_{42}H_{78}O_7N$
DGTA 32:3	706.5622	706.5596	-3.65	14:0_18:3	$C_{42}H_{76}O_7N$
DGTA 32:4	704.5465	704.5455	-1.46	14:0_18:4 and 16:0_16:4	$C_{42}H_{74}O_7N$
DGTA 34:1	738.6248	738.6232	-2.14	16:0_18:1	$C_{44}H_{84}O_7N$
DGTA 34:2	736.6091	736.6083	-1.12	16:0_18:2	$C_{44}H_{82}O_7N$
DGTA 34:3	734.5935	734.5915	-2.69	16:0_18:3	$C_{44}H_{80}O_7N$
DGTA 34:4	732.5778	732.5768	-1.40	16:0_18:4	$C_{44}H_{78}O_7N$
DGTA 34:5	730.5622	730.5620	-0.25	16:1_18:4	$C_{44}H_{76}O_7N$
DGTA 34:6	728.5465	728.5433	-4.43	*	$C_{44}H_{74}O_7N$
DGTA 34:7	726.5309	726.5305	-0.52	**	$C_{44}H_{72}O_7N$
DGTA 36:1	766.6561	766.6528	-4.28	**	$C_{46}H_{88}O_7N$
DGTA 36:2	764.6404	764.6399	-0.69	18:1/18:1	$C_{46}H_{86}O_7N$
DGTA 36:3	762.6248	762.6219	-3.78	**	$C_{46}H_{84}O_7N$
DGTA 36:4	760.6091	760.6069	-2.93	**	$C_{46}H_{82}O_7N$
DGTA 36:5	758.5935	758.5923	-1.55	16:0_20:5	$C_{46}H_{80}O_7N$
DGTA 36:6	756.5778	756.5761	-2.29	**	$C_{46}H_{78}O_7N$
DGTA 36:7	754.5622	754.5594	-3.68	*	$C_{46}H_{76}O_7N$
DGTA 36:8	752.5465	752.5432	-4.42	**	$C_{46}H_{74}O_7N$
DGTA 38:1	794.6874	794.6881	0.91	**	$C_{48}H_{92}O_7N$

DGTA 38:5	786.6248	786.6229	-2.39	18:1_20:4	$C_{48}H_{84}O_7N$
DGTA 38:6	784.6091	784.6072	-2.46	18:2_20:4	$C_{48}H_{82}O_7N$
DGTA 38:7	782.5935	782.5913	-2.78	*	$C_{48}H_{80}O_7N$
DGTA 38:8	780.5778	780.5796	2.27	**	$C_{48}H_{78}O_7N$
DGTA 40:6	812.6404	812.6406	0.21	18:2_22:4	$C_{50}H_{86}O_7N$
DGTA 40:7	810.6248	810.6237	-1.33	**	$C_{50}H_{84}O_7N$
DGTA 40:8	808.6091	808.6075	-2.01	*	$C_{50}H_{82}O_7N$
DGTA 40:9	806.5935	806.5912	-2.83	*	$C_{50}H_{80}O_7N$
DGTA 40:10	804.5778	804.5748	-3.76	**	$C_{50}H_{78}O_7N$
DGTA 42:5	842.6874	842.6871	-0.33	**	$C_{52}H_{92}O_7N$
DGTA 42:11	830.5935	830.5896	-4.67	**	$C_{52}H_{80}O_7N$
**MGTA identified as $[M+H]^+$**					
MGTA 14:0	446.3482	446.3472	-2.16	14:0	$C_{24}H_{48}O_6N$
MGTA 16:0	474.3795	474.3786	-1.82	*	$C_{26}H_{48}O_6N$
MGTA 16:1	472.3638	472.3632	-1.30	*	$C_{26}H_{50}O_6N$
MGTA 18:1	500.3951	500.3945	-1.23	*	$C_{28}H_{54}O_6N$
MGTA 18:2	498.3795	498.3788	-1.33	*	$C_{28}H_{52}O_6N$
MGTA 18:3	496.3638	496.3620	-3.65	18:3	$C_{28}H_{50}O_6N$
MGTA 18:4	494.3482	494.3478	-0.74	**	$C_{28}H_{48}O_6N$
MGTA 20:1	528.4264	528.4265	0.16	*	$C_{30}H_{58}O_6N$
MGTA 20:2	526.4108	526.4101	-1.26	**	$C_{30}H_{56}O_6N$
MGTA 20:3	524.3951	524.3950	-0.22	**	$C_{30}H_{54}O_6N$
MGTA 20:4	522.3795	522.3779	-2.99	*	$C_{30}H_{52}O_6N$
MGTA 20:5	520.3638	520.3630	-1.56	**	$C_{30}H_{50}O_6N$
MGTA 22:1	556.4577	556.4580	0.51	*	$C_{32}H_{62}O_6N$
